# Clearance of damaged mitochondria via mitophagy is important to the protective effect of ischemic preconditioning in kidneys

**DOI:** 10.1080/15548627.2019.1615822

**Published:** 2019-05-22

**Authors:** Man J. Livingston, Jinghong Wang, Jiliang Zhou, Guangyu Wu, Ian G. Ganley, Joseph A. Hill, Xiao-Ming Yin, Zheng Dong

**Affiliations:** a Department of Cellular Biology and Anatomy, Augusta University and Charlie Norwood VA Medical Center, Augusta, GA, USA; b Departments of Laboratory Medicine and Nephrology The Second Xiangya Hospital, Central South University, Changsha, China; c Department of Pharmacology and Toxicology, Medical College of Georgia, Augusta University and Charlie Norwood VA Medical Center, Augusta, GA, USA; d Medical Research Council Protein Phosphorylation and Ubiquitylation Unit, University of Dundee, Dundee, Scotland, UK; e Division of Cardiology, Departments of Internal Medicine and Molecular Biology, University of Texas Southwestern Medical Center, Dallas, TX, USA; f Department of Pathology and Laboratory Medicine, Indiana University School of Medicine, Indianapolis, IN, USA

**Keywords:** Acute kidney injury, autophagy, ischemic preconditioning, mitophagy, proximal tubule, renal ischemia-reperfusion

## Abstract

Ischemic preconditioning (IPC) affords tissue protection in organs including kidneys; however, the underlying mechanism remains unclear. Here we demonstrate an important role of macroautophagy/autophagy (especially mitophagy) in the protective effect of IPC in kidneys. IPC induced autophagy in renal tubular cells in mice and suppressed subsequent renal ischemia-reperfusion injury (IRI). The protective effect of IPC was abolished by pharmacological inhibitors of autophagy and by the ablation of *Atg7* from kidney proximal tubules. Pretreatment with BECN1/Beclin1 peptide induced autophagy and protected against IRI. These results suggest the dependence of IPC protection on renal autophagy. During IPC, the mitophagy regulator PINK1 (PTEN induced putative kinase 1) was activated. Both IPC and BECN1 peptide enhanced mitolysosome formation during renal IRI in mitophagy reporter mice, suggesting that IPC may protect kidneys by activating mitophagy. We further established an in vitro model of IPC by inducing ‘chemical ischemia’ in kidney proximal tubular cells with carbonyl cyanide 3-chlorophenylhydrazone (CCCP). Brief treatment with CCCP protected against subsequent injury in these cells and the protective effect was abrogated by autophagy inhibition. In vitro IPC increased mitophagosome formation, enhanced the delivery of mitophagosomes to lysosomes, and promoted the clearance of damaged mitochondria during subsequent CCCP treatment. IPC also suppressed mitochondrial depolarization, improved ATP production, and inhibited the generation of reactive oxygen species. Knockdown of *Pink1* suppressed mitophagy and reduced the cytoprotective effect of IPC. Together, these results suggest that autophagy, especially mitophagy, plays an important role in the protective effect of IPC.

**Abbreviations**: ACTB: actin, beta; ATG: autophagy related; BNIP3: BCL2 interacting protein 3; BNIP3L/NIX: BCL2 interacting protein 3 like; BUN: blood urea nitrogen; CASP3: caspase 3; CCCP: carbonyl cyanide 3-chlorophenylhydrazone; COX4I1: cytochrome c oxidase subunit 4I1; COX8: cytochrome c oxidase subunit 8; DAPI: 4ʹ,6-diamidino-2-phenylindole; DNM1L: dynamin 1 like; EGFP: enhanced green fluorescent protein; EM: electron microscopy; ER: endoplasmic reticulum; FC: floxed control; FIS1: fission, mitochondrial 1; FUNDC1: FUN14 domain containing 1; H-E: hematoxylin-eosin; HIF1A: hypoxia inducible factor 1 subunit alpha; HSPD1: heat shock protein family D (Hsp60) member 1; IMMT/MIC60: inner membrane mitochondrial protein; IPC: ischemic preconditioning; I-R: ischemia-reperfusion; IRI: ischemia-reperfusion injury; JC-1: 5,5ʹ,6,6ʹ-tetrachloro-1,1ʹ,3,3ʹ-tetraethylbenzimidazolylcarbocyanine iodide; KO: knockout; MAP1LC3B/LC3B: microtubule associated protein 1 light chain 3 beta; mito-QC: mito-quality control; mRFP: monomeric red fluorescent protein; NAC: N-acetylcysteine; PINK1: PTEN induced putative kinase 1; PPIB: peptidylprolyl isomerase B; PRKN: parkin RBR E3 ubiquitin protein ligase; ROS: reactive oxygen species; RPTC: rat proximal tubular cells; SD: standard deviation; sIPC: simulated IPC; SQSTM1/p62: sequestosome 1; TOMM20: translocase of outer mitochondrial membrane 20; TUNEL: terminal deoxynucleotidyl transferase-mediated dUTP nick end labeling

## Introduction

Ischemic preconditioning (IPC) consists of a short period of non-lethal ischemia-reperfusion (I-R), which is one of the most effective interventions known to protect the heart, brain, kidney, liver, and other organs from subsequent severe ischemia-reperfusion injury (IRI) [1,2]. From studies in myocardium, two windows of protection are characterized in IPC: an early effect termed classical or acute IPC and a late phase of resistance known as second window of protection or delayed IPC. Acute IPC occurs immediately following the preconditioning stimulus and lasts for 2 to 3 h. Delayed IPC reappears a day later and lasts for a few days [1]. In the kidney, IPC has been shown to reduce acute kidney injury induced by renal I-R in many animal species [3–5]. Renal IPC is also effective in both the early and late phases of preconditioning [6]. As an endogenous defense mechanism, IPC induces a number of adaptive changes in the preconditioned tissues, including altered energy metabolism, better electrolyte and acid-base homeostasis, less production of oxygen free radicals, reduced inflammatory responses and apoptosis, and improved microcirculatory perfusion. All of these changes contribute to the tolerance of the preconditioned tissues to sustained ischemia and following reperfusion [2].

Autophagy is an adaptive response activated upon stress to maintain cellular energy homeostasis and to remove protein aggregates and damaged organelles via the autolysosomal degradation pathway [7,8]. Growing evidence has suggested the involvement of autophagy in IPC protection in the heart, brain and liver. IPC induces autophagy in myocardium of mice and in isolated rat hearts, which is essential for the cardioprotection afforded by IPC [9,10]. In rat brains, autophagy is activated by focal cerebral IPC and protects against brain damage induced by subsequent permanent focal ischemia [11–14]. In addition, IPC in livers protects against IRI via autophagy mediated by heme oxygenase-1 or nitric oxide [15,16]. Despite these findings, whether autophagy contributes to the protection of IPC in kidneys has not been reported. Moreover, it remains unclear how autophagy in IPC protects against subsequent tissue damage.

Maintaining a healthy mitochondrial network is critical for cell survival, as accumulation of damaged mitochondria produces reactive oxygen species (ROS) and also releases cell death factors. The selective clearance of damaged mitochondria by autophagy is termed mitophagy, which serves an endpoint of mitochondrial quality control [17–19]. Mitophagy initiation requires: (1) mitochondrial fragmentation to produce size-suitable mitochondria for autophagic sequestration; (2) priming of the dysfunctional mitochondria for autophagy recognition; (3) induction of general autophagy machinery to incorporate the primed mitochondria into autophagosomes. Among these, the priming of mitochondria for clearance is a distinct stage and regulated by coordination and interplay of multiple signaling pathways [17–19]. The best characterized mitophagy pathway to date is PINK1 (PTEN induced putative kinase 1)- PRKN (parkin RBR E3 ubiquitin protein ligase) pathway. PINK1 senses mitochondrial damage, accumulates on the outer mitochondrial membrane, and recruits cytosolic PRKN to mitochondria. Once recruited, PRKN ubiquitinates outer mitochondrial membrane proteins to enable recognition of the marked mitochondria by receptor proteins such as SQSTM1/p62 (sequestosome 1) for autophagic elimination [20]. The association of mitophagy with IPC protection is largely unknown. Nonetheless, PRKN-mediated mitophagy has been implicated in IPC-mediated cardioprotection in Langendorff-perfused rat hearts [21].

We and others recently have demonstrated a protective role of autophagy in renal IRI [22–26]. Using *Pink1* and *Prkn* single- as well as double-knockout mouse models, our latest work has further suggested a protective role of PINK1-PRKN-mediated mitophagy against renal IRI [27]. The current study sought to examine tubular cell autophagy in renal IPC. We showed that autophagy was activated in proximal tubules by renal IPC in mice. Impairment of autophagy either by pharmacological inhibitors or in proximal tubule-specific *atg7* knockout (PT-*atg7* KO) mice compromised the beneficial effects of renal IPC, whereas preconditioning with autophagy-inducing Tat-BECN1/Beclin1 peptide afforded IPC-like renoprotection. In cultured proximal tubular cells, the cytoprotection of in vitro simulated IPC (sIPC) was also abolished by autophagy inhibition. These results demonstrate that autophagy in proximal tubules plays an essential role in the renoprotection of IPC. Mechanistically, IPC promoted mitophagy likely via the PINK1-PRKN pathway. Enhanced clearance of damaged mitochondria attenuated mitochondrial dysfunction and ROS generation, thus preventing tubular cell apoptosis and subsequent renal IRI.

## Results

### Renal IPC protects against renal IRI in C57BL/6 mice

**Figure 1.**
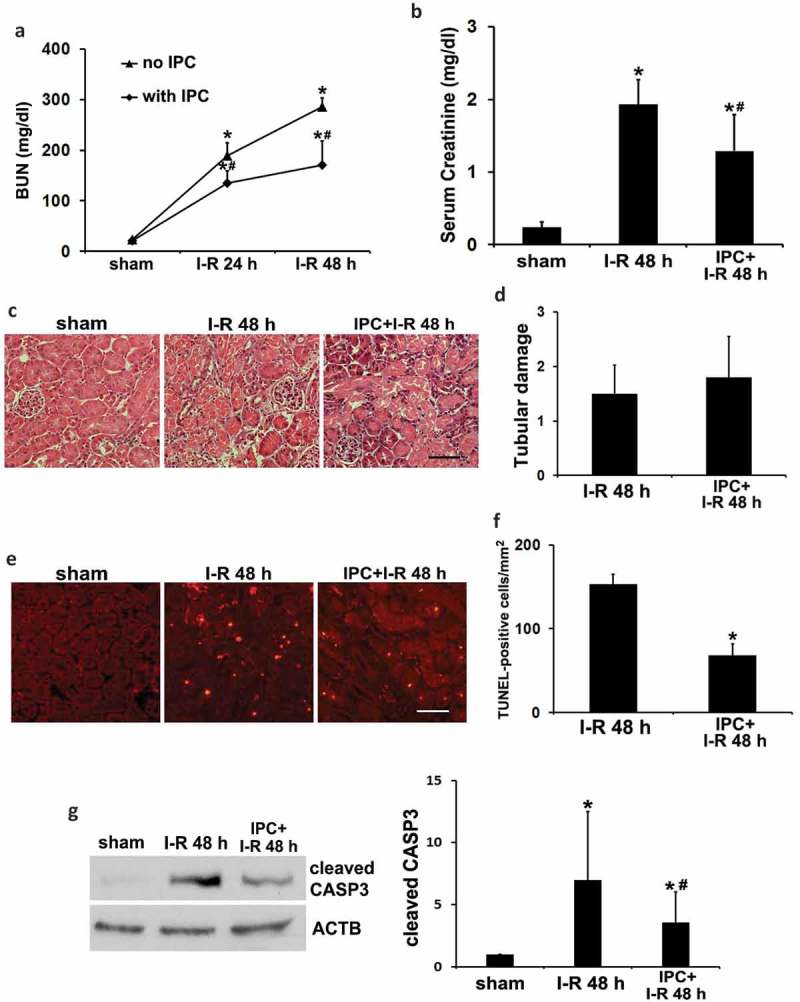
Renal IPC protects against renal IRI in C57BL/6 mice. Mice were subjected to 15-min bilateral renal ischemia followed by 1 h of reperfusion to induce renal IPC. To induce renal IRI, mice were subsequently treated with 27-min bilateral renal ischemia followed by 24 to 48 h of reperfusion (I-R) (sham: n = 3; I-R: n = 7; IPC + I-R: n = 10). Blood and kidneys were collected at the indicated time points for renal function, histology and immunoblot analyses. (a) BUN. (b) Serum creatinine. Data are expressed as mean ± SD. *, *P* < 0.05, significantly different from the sham group; #, *P* < 0.05, significantly different from I-R group. (c) Representative histology of renal cortex and outer medulla H-E staining. Scale bar: 50 µm. (d) Pathological score of tubular damage. (e) Representative images of TUNEL staining. Scale bar: 50 µm. (f) Quantification of TUNEL-positive cells. Data in (d and f) are expressed as mean ± SD. *, *P* < 0.05, significantly different from I-R group. (g) Representative blots and densitometric analysis of cleaved CASP3. ACTB was used as a loading control. After normalization with ACTB, the protein signal of the sham sample was arbitrarily set as 1, and the signals of other conditions were normalized to the sham to calculate fold changes. Data are expressed as mean ± SD. *, *P* < 0.05, significantly different from the sham group; #, *P* < 0.05, significantly different from I-R group.

Renal IPC was induced in mice by a brief bilateral renal ischemia of 15 min followed by 1 h of reperfusion. Mice were then subjected to a prolonged (27 min) bilateral renal ischemia followed by reperfusion for up to 48 h to examine renal IRI. In functional analysis, renal IRI alone induced severe injury as indicated by increases of blood urea nitrogen (BUN) and serum creatinine to 286 mg/dl and 1.93 mg/dl, respectively (Figure 1(a,b), I–R). IPC partially yet significantly reduced BUN and serum creatinine to 171 mg/dl and 1.29 mg/dl (Figure 1(a,b), IPC + I-R vs I-R). In histological analysis by hematoxylin-eosin (H-E) staining, renal IRI led to necrotic tubular cell death in kidney cortex and outer medulla, which was not significantly affected by renal IPC (Figure 1(c,d)). In sharp contrast, terminal deoxynucleotidyl transferase-mediated dUTP nick end labeling (TUNEL) staining showed that renal IPC markedly attenuated renal tubule apoptosis in IRI (Figure 1(e)). Quantitatively, the number of TUNEL-positive cells per mm^2^ tissue was reduced from 153 to 68 by IPC (Figure 1(f)). In support of the TUNEL results, renal IRI induced cleaved/active CASP3 (caspase 3) in kidneys, which was significantly suppressed by renal IPC (Figure 1(g)). Together, these results confirm the renoprotective effects of IPC in our experimental settings. The inhibitory effects of IPC on tubular cell apoptosis may mainly account for the improvement of renal function.

### Induction of autophagy in proximal tubules during renal IPC

**Figure 2.**
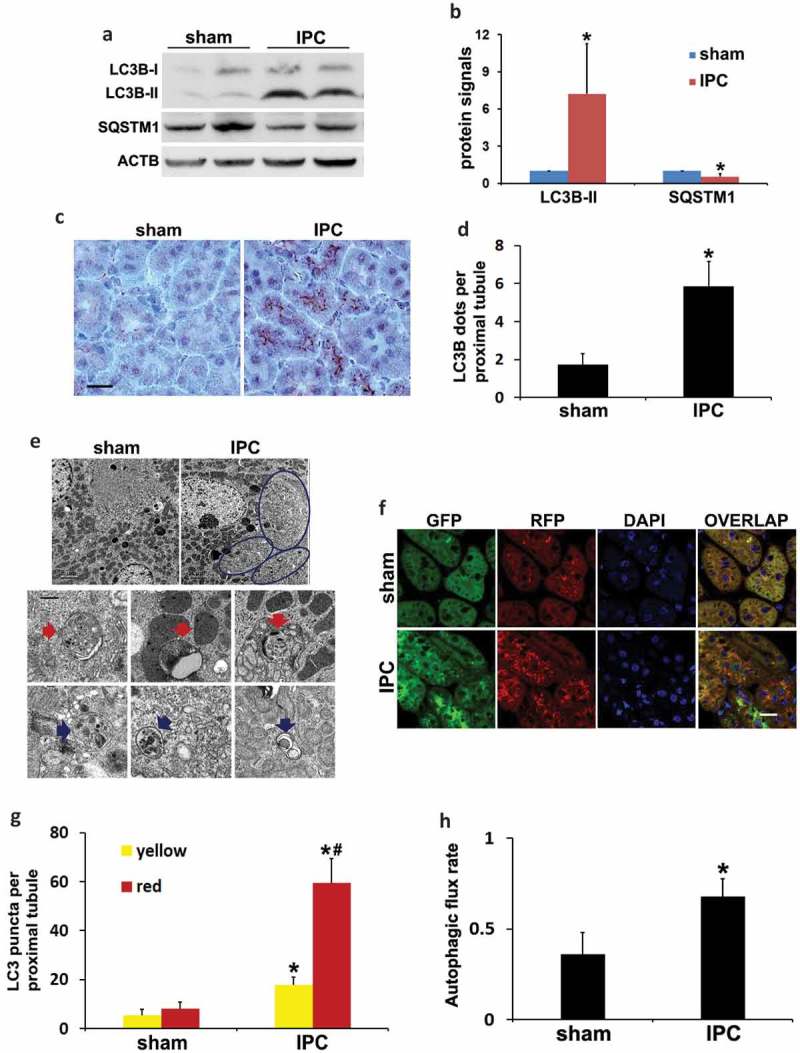
Induction of autophagy in proximal tubules during renal IPC. C57BL/6 (a–e) and CAGp-RFP-GFP-LC3 (f–h) mice were subjected to renal IPC only without subsequent renal IRI (C57BL/6 mice: sham: n = 3; IPC: n = 6; CAGp-RFP-GFP-LC3 mice: n = 3 for each). Kidneys were collected after preconditioning for histological and immunoblot analyses. (a) Representative blots of LC3B and SQSTM1. ACTB was used as a loading control. (b) Densitometric analysis of LC3B-II and SQSTM1. After normalization with ACTB, the protein signals of the sham were arbitrarily set as 1, and the signals of other conditions were normalized to the sham to calculate fold changes. (c) Representative images of immunohistochemical staining of LC3B. Scale bar: 20 µm. (d) Quantitative analysis of punctate LC3B staining. Data in (b and d) are expressed as mean ± SD. *, *P* < 0.05, significantly different from the sham group. (e) Representative electron micrographs showing autophagic vacuoles (arrows) (n = 2 for each). Scale bar: 2 µm (3 upper panels) and 200 nm (6 lower panels). (f) Representative images of GFP-LC3 and RFP-LC3 fluorescence staining. Scale bar: 15 µm. (g) Quantitative analysis of yellow and red LC3 puncta. Data are expressed as mean ± SD. *, *P* < 0.05, significantly different from the sham group; #, *P* < 0.05, values of red LC3 puncta significantly different from the relevant values of yellow LC3 puncta. (h) Analysis of autophagic flux rate. Data are expressed as mean ± SD. *, *P* < 0.05, significantly different from the sham group.

We next determined autophagy induction during renal IPC using multiple methods. IPC in mice induced an accumulation of the lipidated form of MAP1LC3B/LC3B (microtubule associated protein 1 light chain 3 beta; LC3B-II) and degradation of SQSTM1 in kidneys (Figure 2(a,b)). Immunohistochemical staining of LC3B revealed a granular LC3B staining especially along the brush boarder of proximal tubules in preconditioned kidneys, suggesting the formation of autophagosomes (Figure 2(c)). By quantification, the number of LC3B dots per proximal tubule was increased from 2 in sham group to 6 by renal IPC (Figure 2(d)). Electron microscopy (EM) confirmed that autophagic vacuoles induced by IPC were mainly seen at the apical side of proximal tubular cells underneath the microvilli (Figure 2(e), circled areas). The structures of typical autophagic vacuoles were shown at high magnification (10,000×) EM (Figure 2(e)). Autophagosomes appeared as double-membrane structures containing cytoplasmic materials and/or organelles (red arrows). Autolysosomes were identified as single-membrane structures containing cytoplasmic components at various stages of degradation (blue arrows). Along with increased autophagy, the number of reabsorption vacuoles was remarkably decreased by renal IPC. Sham mice had numerous reabsorption vacuoles in the apical cytoplasm of proximal tubular cells, including large vacuoles and small coated vesicles, which were rarely seen in preconditioned kidneys (Figure S1). In addition to the steady-state LC3B staining and EM images, we further monitored the dynamics of autophagy using CAGp-RFP-GFP-LC3 transgenic mice. These autophagy reporter mice express a tandem RFP-GFP-LC3 fusion protein ubiquitously under the control of a *CAG* promoter [28]. The dual-color fluorescent probe utilizes the differential stabilities of RFP and GFP in the lysosome to discriminate between autophagosomes and autolysosomes. RFP signal is typically maintained despite the acidic lysosomal environment (pH 4 to 5) whereas GFP loses fluorescence in this pH range. Therefore, colocalization of GFP with RFP fluorescence in a particle indicates an autophagosome and a RFP-only signal without GFP is considered an autolysosome [28,29]. Using this mouse model, our recent work has demonstrated the dynamic changes of autophagy in proximal tubules during kidney fibrosis induced by obstructive uropathy [30]. In the current study, compared to sham control that had minimal amounts of GFP-LC3 and RFP-LC3 puncta indicating a low basal level of autophagy, renal IPC significantly induced the formation of autophagosomes as well as the maturation to autolysosomes in proximal tubules (Figure 2(f)). Quantification of LC3 puncta showed that the number of autophagosomes per proximal tubule was increased from 5 in sham mice to 18 by renal IPC (Figure 2(g), yellow). There were 3 autolysosomes per proximal tubule in the sham group, which was increased to 41 in preconditioned mice (Figure 2(g), red-only). We then calculated the autophagic flux rate using the number of autolysosomes divided by the total number of red puncta. Sham mice had a basal rate of 36% autophagic flux, which was significantly increased to 68% by IPC (Figure 2(h)). These results provide definite evidence that autophagy is induced in proximal tubules following renal IPC. We further monitored the effects of IPC on autophagy during subsequent renal IRI. Unlike the studies in the heart and brain [9,11], renal IPC did not change the induction of general autophagy during prolonged ischemia in the kidney. Mice in renal IRI group and in IPC + renal IRI group had comparable extents of LC3B-II accumulation and degradation of SQSTM1 (Figure S2(a)). Morphological examination in autophagy reporter mice confirmed that IPC did not further enhance IRI-induced autophagosome formation and maturation to autolysosomes in kidney proximal tubules (Figure S2(b–d)).

## *The renoprotective effects of IPC are abrogated by autophagy inhibitors and in PT-*atg7 *KO mice*

To determine the role of autophagy in IPC renoprotection, C57BL/6 mice were pretreated with IPC and then subjected to renal IRI in the absence or presence of chloroquine or 3-methyladenine, two pharmacological inhibitors of autophagy. The effects of these inhibitors on autophagy were first verified by immunoblot analysis of LC3B and SQSTM1. As shown in Figure S3(a), renal IPC led to LC3B-II accumulation and SQSTM1 degradation. By preventing autolysosomal acidification, chloroquine enhanced LC3B-II accumulation and prevented SQSTM1 degradation. 3-methyladenine, via inhibiting autophagosome formation, reduced both LC3B-II accumulation and SQSTM1 degradation. Similarly, these inhibitors also suppressed autophagy activation during subsequent renal IRI (Figure S3(b and c)). We then examined the effects of chloroquine and 3-methyladenine on kidney injury. In the absence of the inhibitors, IPC prevented renal function loss, as indicated by the decreases of BUN and serum creatinine (Figure S3(d and e), saline). Both chloroquine and 3-methyladenine abolished the beneficial effects of renal IPC (Figure S3(d and e)), suggesting that autophagy is indispensable for IPC renoprotection during renal IRI.

**Figure 3.**
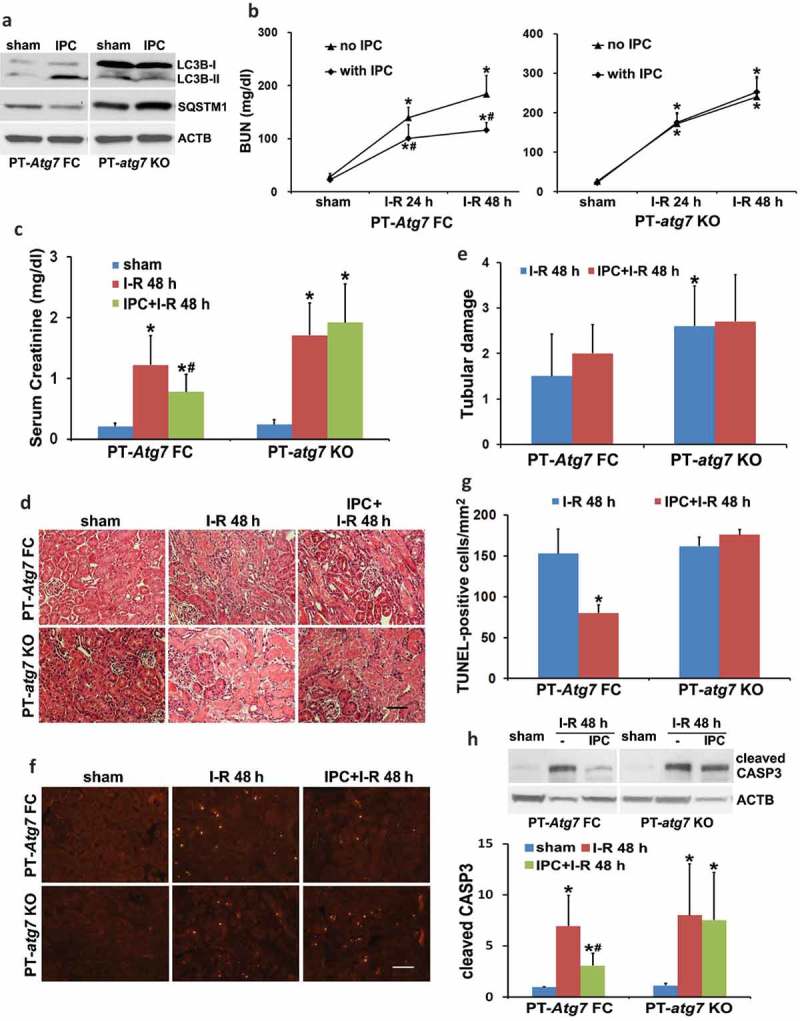
The protective effects of IPC against renal IRI are abrogated in PT-*atg7* KO mice. (a) Floxed control (PT-*Atg7* FC) and PT-*atg7* KO mice were treated with sham or IPC and kidneys were collected for immunoblot analysis of LC3B and SQSTM1 (n = 3 for each). ACTB was used as a loading control. Floxed control and PT-*atg7* KO mice were subjected to: (1) sham (n = 3 for each); (2) I-R (n = 8 for FC; n = 9 for KO); (3) IPC + I-R (n = 10 for each). Blood and kidneys were collected at the indicated time points for renal function, histology and immunoblot analyses. (b) BUN. (c) Serum creatinine. Data are expressed as mean ± SD. *, *P* < 0.05, significantly different from the sham group; #, *P* < 0.05, significantly different from I-R group. (d) Representative histology of renal cortex and outer medulla H-E staining. Scale bar: 50 µm. (e) Pathological score of tubular damage. (f) Representative images of TUNEL staining. Scale bar: 50 µm. (g) Quantification of TUNEL-positive cells. Data in (e and g) are expressed as mean ± SD. *, *P* < 0.05, significantly different from I-R group. (h) Representative blots and densitometric analysis of cleaved CASP3. ACTB was used as a loading control. After normalization with ACTB, the protein signal of the sham was arbitrarily set as 1, and the signals of other conditions were normalized to the sham to calculate fold changes. Data are expressed as mean ± SD. *, *P* < 0.05, significantly different from the sham group; #, *P* < 0.05, significantly different from I-R group.

To verify the pharmacological results, we further examined PT-*atg7* KO mice in which *Atg7* was specifically ablated from kidney proximal tubules [23]. The inhibitory effects of *Atg7* deletion on renal IPC-induced autophagy was confirmed by immunoblots of LC3B and SQSTM1 (Figure 3(a)). Renal IRI without IPC led to kidney injury in both floxed control (PT-*Atg7* FC) and PT-a*tg7* KO mice. Consistent with our previous work [23], PT-*atg7* KO mice were more sensitive to the injury as compared to floxed control mice, showing more severe renal function loss and tubular cell death (Figure 3(b–e), no IPC, PT-*atg7* KO vs PT-*Atg7* FC). Renal IPC attenuated the BUN and serum creatinine increases in floxed control mice (Figure 3(b,c), PT-*Atg7* FC), whereas the effect of IPC was completely lost in PT-*atg7* KO mice (Figure 3(b,c), PT-*atg7* KO). Morphologically, IPC was unable to inhibit tubular cell necrosis in both floxed control and PT-*atg7* KO mice (Figure 3(d,e)). Nevertheless, IPC significantly reduced tubular cell apoptosis in floxed control mice and this effect was eliminated in PT-*atg7* KO mice, as shown by the analyses of TUNEL staining and CASP3 cleavage (Figure 3(f–h)). Together, these results provide compelling evidence that autophagy plays an essential role in the protective effect of IPC against subsequent renal IRI.

## Pharmacological preconditioning with Tat-BECN1 activates autophagy and protects against renal IRI in C57BL/6 mice

**Figure 4.**
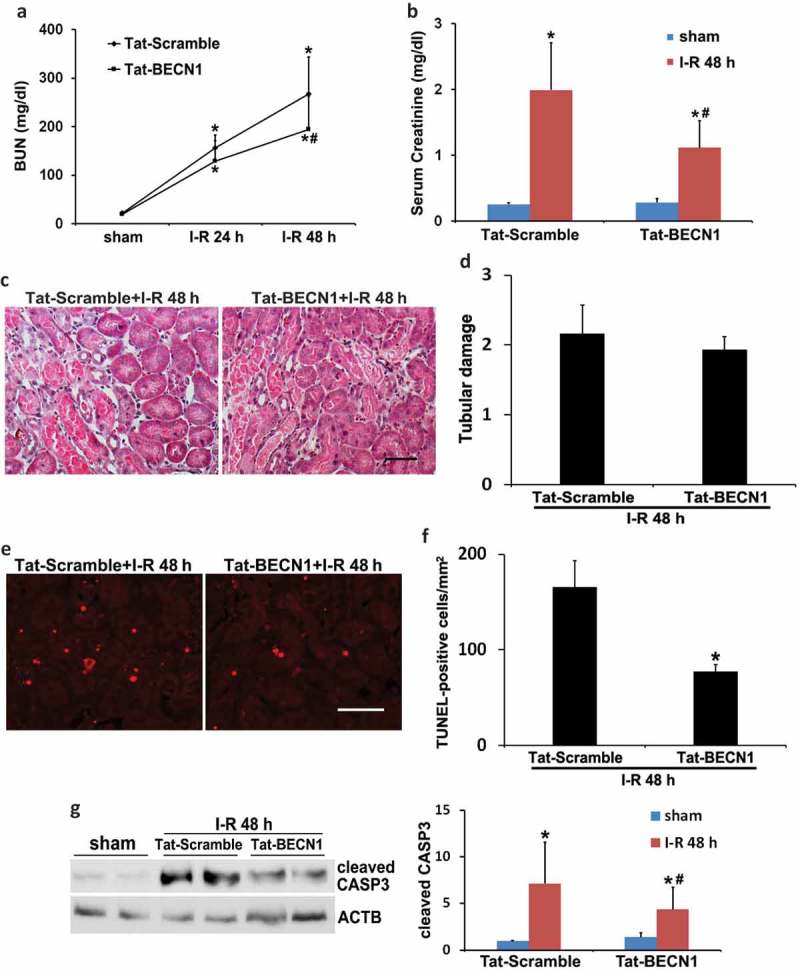
Pharmacological preconditioning with Tat-BECN1 protects against renal IRI in C57BL/6 mice. C57BL/6 mice were pretreated with Tat-BECN1 and its control peptide (Tat-Scramble) at a single dose of 20 mg/kg i.p. injection. Four h after preconditioning, mice were subjected to 27-min bilateral renal ischemia followed by 24 to 48 h of reperfusion (sham: n = 3 for each; Tat-Scramble + I-R: n = 8; Tat-BECN1 + I-R: n = 10). Blood and kidneys were collected at the indicated time points for renal function, histology and immunoblot analyses. (a and b) BUN and serum creatinine. Data are expressed as mean ± SD. *, *P* < 0.05, significantly different from the sham group; #, *P* < 0.05, significantly different from Tat-Scramble + I-R group. (c) Representative histology of renal cortex and outer medulla H-E staining. Scale bar: 50 µm. (d) Pathological score of tubular damage. (e) Representative images of TUNEL staining. Scale bar: 50 µm. (f) Quantification of TUNEL-positive cells. Data in (d and f) are expressed as mean ± SD. *, *P* < 0.05, significantly different from Tat-Scramble + I-R group. (g) Representative blots and densitometric analysis of cleaved CASP3. ACTB was used as a loading control. After normalization with ACTB, the protein signal of Tat-Scramble + sham was arbitrarily set as 1, and the signals of other conditions were normalized to the Tat-Scramble + sham to calculate fold changes. Data are expressed as mean ± SD. *, *P* < 0.05, significantly different from the sham group; #, *P* < 0.05, significantly different from Tat-Scramble + I-R group.

To mimic the effect of renal IPC on autophagy, we pretreated C57BL/6 mice with a recently identified specific autophagy-inducing peptide, Tat-BECN1 [31]. Our pilot test indicated that autophagy was induced in kidneys at 3 to 4 h after Tat-BECN1 injection. We therefore chose to administer the peptide 4 h before the onset of 27-min ischemia and determined its effect on kidney injury. Activation of autophagy by Tat-BECN1 was first indicated by LC3B-II induction and SQSTM1 degradation in pretreated kidneys (Figure S4(a and b)). Immunohistochemical staining of LC3B further revealed that compared to scramble peptide, Tat-BECN1 pretreatment enhanced the autophagosome formation in proximal tubules, with the number of punctate LC3B increased from 2 to 6 per proximal tubule (Figure S4(c and d)). The dynamic changes of autophagy were also determined in autophagy reporter mice. Tat-BECN1 treatment significantly increased the formation of both autophagosomes and autolysosomes (Figure S4(e and f)). Consistently, autophagic flux was also elevated by Tat-BECN1 (Figure S4(g)). These results suggest that pharmacological preconditioning with Tat-BECN1 activates autophagy in kidney proximal tubules. We then monitored kidney injury. Mice pretreated with Tat-BECN1 showed significantly lower BUN and serum creatinine during renal IRI as compared to scramble peptide-injected group (Figure 4(a,b)). Similar with the effects of renal IPC, Tat-BECN1 preconditioning did not prevent tubular necrosis (Figure 4(c,d)). Nevertheless, the peptide remarkably suppressed tubular apoptosis in renal IRI. The number of TUNEL-positive cells was reduced from 166 in Tat-Scramble group to 78 by Tat-BECN1, which also reduced the cleavage or activation of CASP3 (Figure 4(e–g)). These results further support an important role of autophagy in the protective effect of preconditioning. Pharmacological upregulation of autophagy (pretreatment) may be an effective approach for the prevention and treatment of renal IRI.

## Both renal IPC and Tat-BECN1 preconditioning enhance mitophagy in proximal tubules during subsequent renal IRI in mice

**Figure 5.**
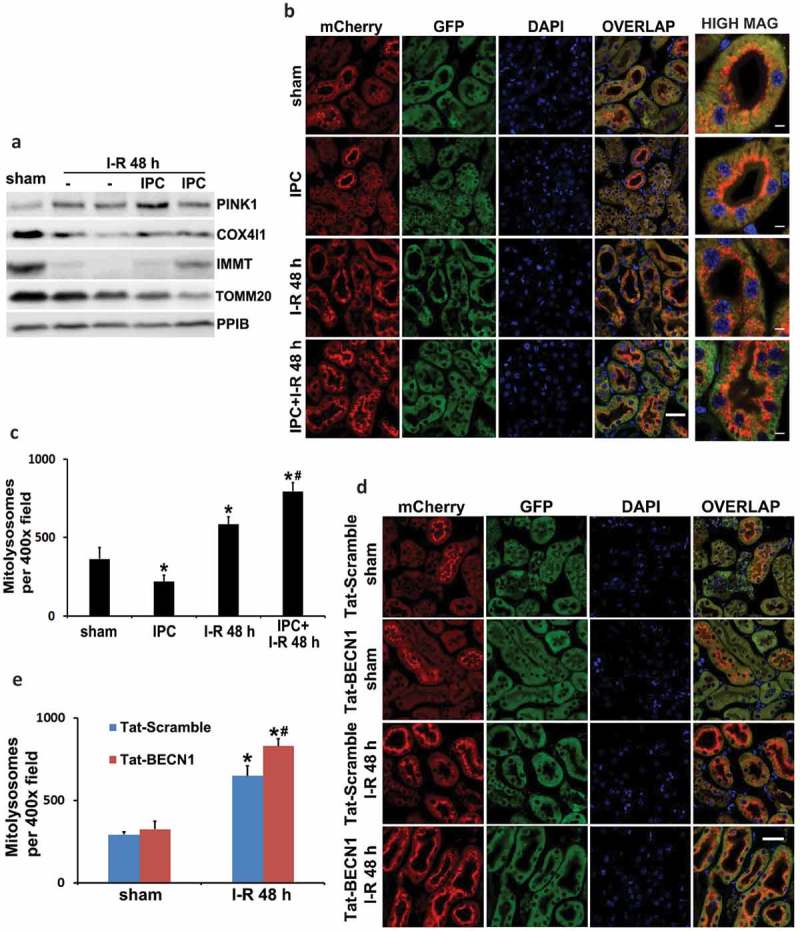
Both renal IPC and Tat-BECN1 preconditioning enhance mitophagy in proximal tubules during subsequent renal IRI in mice. (a) C57BL/6 mice were subjected to: (1) sham (n = 3); (2) I-R (n = 7); (3) IPC + I-R (n = 10). Kidneys were collected for immunoblot analysis of PINK1, COX4I1, IMMT/MIC60, and TOMM20. PPIB (peptidylprolyl isomerase B) was used as a loading control. Mito-QC mice were subjected to: (1) sham (n = 3); (2) IPC (n = 3); (3) I-R (n = 4); (4) IPC + I-R (n = 5). Kidneys were collected to determine mitophagy flux by fluorescence microscopy. (b) Representative images of the formation of mitolysosomes. Scale bar: 20 µm for low magnification and 5 µm for high magnification. (c) Quantitative analysis of the number of mitolysosomes per 400× field (renal cortex and outer stripe of outer medulla, glomeruli excluded). Data are expressed as mean ± SD. *, *P* < 0.05, significantly different from the sham group; #, *P* < 0.05, significantly different from I-R group. Furthermore, mito-QC mice were pretreated with Tat-BECN1 and its control peptide (Tat-Scramble) at a single dose of 20 mg/kg i.p. injection. Four h after preconditioning, mice were subjected to sham surgery or 27-min bilateral renal ischemia followed by 48 h of reperfusion (n = 3 for each). Kidneys were collected to determine mitophagy flux by fluorescence microscopy. (d) Representative images of the formation of mitolysosomes. Scale bar: 20 µm. (e) Quantitative analysis of the number of mitolysosomes per 400× field (renal cortex and outer stripe of outer medulla, glomeruli excluded). Data are expressed as mean ± SD. *, *P* < 0.05, significantly different from the sham group; #, *P* < 0.05, significantly different from Tat-Scramble + I-R group.

Removal of protein aggregates and damaged organelles by autophagy is critical for autophagy-dependent protection under various stress conditions. In the heart, mitochondria are the important targets of autophagy elimination (mitophagy) during ischemia-reperfusion or oxidative stress [32,33]. To understand how autophagy in IPC protects kidneys, we examined the effects on mitophagy. As shown in Figure 5(a), PINK1, an important mitophagy regulator that promotes the recruitment of PRKN on the surface of damaged mitochondria to initiate mitophagy, was accumulated in kidneys following renal IRI. Activation of PINK1 was accompanied with loss of mitochondrial mass, as indicated by the decreases of multiple mitochondrial proteins including inner membrane COX4I1 (cytochrome c oxidase subunit 4I1) and IMMT/MIC60 (inner membrane mitochondrial protein) as well as outer membrane TOMM20 (translocase of outer mitochondrial membrane 20) (Figure 5(a), I-R)). Due to the low sensitivity of this biochemical analysis of mitophagy, renal IPC did not show obvious effects on the loss of mitochondrial mass (Figure 5(a), IPC + I-R vs I-R).

To further measure mitophagy and distinguish between different cell types within the kidney, we utilized the newly established mitophagy reporter mouse model named mito-QC (mito-quality control) [34]. Mito-QC mice express a tandem mCherry-GFP protein fused to the mitochondrial targeting sequence of FIS1 (fission, mitochondrial 1) that is pH sensitive. Under steady state, mito-QC displays both red (mCherry) and green (GFP) fluorescence, giving a yellow staining of mitochondrial networks. Upon mitophagy, mitochondria are delivered to lysosomes where GFP signal is quenched in the acidic environment but mCherry fluorescence is stable and maintained. Therefore, the appearance of mCherry-only puncta that colocalize with LAMP1 (lysosomal-associated membrane protein 1) indicates the formation of mitolysosomes. This assay allows a binary, endpoint readout of mitophagy and assessment of mitochondrial architecture in vivo [34,35]. Using this mouse model, we analyzed mitophagy in kidney tissues under control, preconditioning, and renal IRI conditions. Notably, sham control mice had a high basal level of mitophagy in some cortical proximal tubules. In these renal tubules, mitolysosomes displayed a polarized distribution and positioned predominantly at the apical side toward the lumen (Figure 5(b), sham, mCherry). In contrast to these highly mitophagic proximal tubules in renal cortex, proximal tubules in the inner cortex and the outer stripe of outer medulla (S3 segment that is highly susceptible to renal IRI) showed minimal mitochondrial turnover under basal condition (Figure S5(a and b)). Mitophagy in glomeruli was also much less than those cortical proximal tubules (Figure S5(b)). Following renal IRI, mitophagy was significantly induced in proximal tubules. More proximal tubules in both renal cortex and S3 segment had apical-positioned mitolysosomes as compared to sham mice (Figure 5(b), I-R, mCherry). The polarized distribution and increased formation of mitolysosomes were further revealed in individual proximal tubules at a higher magnification (Figure 5(b), high mag). Importantly, renal IPC did not induce mitophagy on its own (Figure 5(b), IPC, mCherry) but further enhanced the intensity of mitolysosome formation in renal IRI (Figure 5(b), IPC + I-R, mCherry). The numbers of mitolysosomes per 400× field (cortex and outer stripe of outer medulla, glomeruli excluded) were counted for quantification. Sham control mice had an average of 364 mitolysosomes, which was increased to 583 in I-R mice and further elevated to 793 in IPC + I-R group (Figure 5(c)). In addition to renal IPC, pharmacological preconditioning with Tat-BECN1 also enhanced mitolysosome formation and mitophagy flux in kidney proximal tubules of mito-QC mice. In sham control animals, Tat-BECN1 pretreatment had a marginal effect, increasing the number of mitolysosomes from 293 to 325 (Figure 5(d,e), Tat-BECN1 + sham vs. Tat-Scramble + sham). Following renal IRI, 650 mitolysosomes were detected in scramble peptide-pretreated group (Figure 5(d,e), Tat-Scramble + I-R), which was significantly elevated to 830 by Tat-BECN1 preconditioning (Figure 5(d,e), Tat-BECN1 + I-R). Together, these results suggest that renal IPC may protect the kidney by promoting mitophagy in proximal tubules during subsequent renal IRI.

### In vitro sIPC attenuates prolonged CCCP-induced apoptosis in renal proximal tubular cells

**Figure 6.**
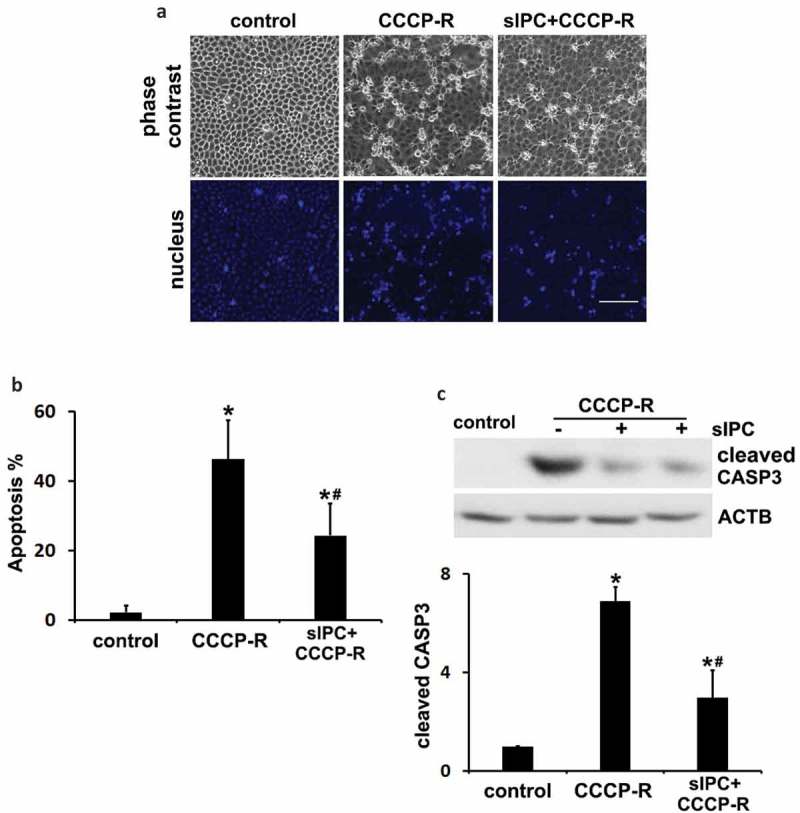
In vitro sIPC attenuates prolonged CCCP-induced apoptosis in RPTC cells. In vitro sIPC was induced by incubating RPTC cells with 20 μM CCCP for 30 min followed by 40 min of recovery. The cells were then treated with prolonged CCCP (20 μM) for 3 h followed by 2 h of recovery to model in vivo renal IRI. Cells were collected for analysis of apoptosis by morphology and caspase activation. (a) Representative images of phase contrast and fluorescence microscopy showing cellular and nuclear morphology of apoptosis. Scale bar: 200 μm. (b) Quantification of cell apoptosis. (c) Representative blots and densitometric analysis of cleaved CASP3. ACTB was used as a loading control. After normalization with ACTB, the protein signal of the control was arbitrarily set as 1, and the signals of other conditions were normalized to the control to calculate fold changes. Data in (b and c) are expressed as mean ± SD. *, *P* < 0.05, significantly different from the control group; #, *P* < 0.05, significantly different from CCCP-R group.

To further delineate the role of autophagy/mitophagy in IPC renoprotection, we established an in vitro model where sIPC was induced in rat proximal tubular cells (RPTC) by 30 min of ATP-depletion with carbonyl cyanide 3-chlorophenylhydrazone (CCCP) followed by 40 min of recovery. The cells were then incubated with prolonged CCCP for 3 h followed by 2 h of recovery to model in vivo renal IRI. The cytoprotective effects of sIPC in cultured proximal tubular cells were verified by morphological and biochemical analyses. Prolonged CCCP treatment led to 46% apoptosis in RPTC cells, which was significantly reduced to 24% by sIPC. The apoptotic cells displayed a shrunken configuration with apoptotic bodies and condensed or fragmented nuclei (Figure 6(a,b)). Consistently, CCCP-induced caspase activation was also attenuated by sIPC. There was a 7-fold increase of cleaved CASP3 expression in prolonged CCCP-treated cells, which was reduced to 3-fold by sIPC (Figure 6(c)). In addition to CCCP, we also used sodium azide for ATP depletion to mimic in vivo IPC and IRI. Consistently, sIPC suppressed azide-induced apoptosis in RPTC cells (Figure S6).

### Autophagy is activated by in vitro sIPC in RPTC cells

We then examined autophagy in preconditioned RPTC cells. As shown in Figure S7(a), sIPC induced both LC3B-II accumulation and SQSTM1 degradation in RPTC cells, suggesting an activation of autophagy under this condition. Upregulation of LC3B-II was further enhanced in the presence of chloroquine, indicating an increased LC3B-II turnover and induction of autophagic flux (Figure S7(b)). The dynamic changes of autophagy were further monitored in RPTC cells transfected with a tandem fluorescent-tagged LC3 (mRFP-GFP-LC3). Control cells had a very low basal level of autophagy as indicated by minimal punctate staining (Figure S7(c), control). sIPC increased the formation of both autophagosomes and autolysosomes (Figure S7(c), sIPC). Quantitatively, the number of autophagosomes (yellow puncta) per cell was increased from 6 in control cells to 15 in preconditioned cells. The number of autolysosomes (red-only puncta) per cell was also remarkably elevated from 2 in control group to 18 by sIPC (Figure S7(d)). Accordingly, autophagic flux rate was increased from 27% in control to 54% by sIPC (Figure S7(e)). Together, these results suggest that autophagy is induced in preconditioned RPTC cells. Notably, consistent with in vivo findings, in vitro sIPC did not have additional effects on general autophagy induced during subsequent CCCP treatment of RPTC cells, as shown by immunoblot analysis of LC3B and SQSTM1 (Figure S8(a)) as well as morphological examination of autophagosomes, autolysosomes and autophagic flux (Figure S8(b–d)).

### The cytoprotection of in vitro sIPC is diminished by autophagy inhibitors in RPTC cells

**Figure 7.**
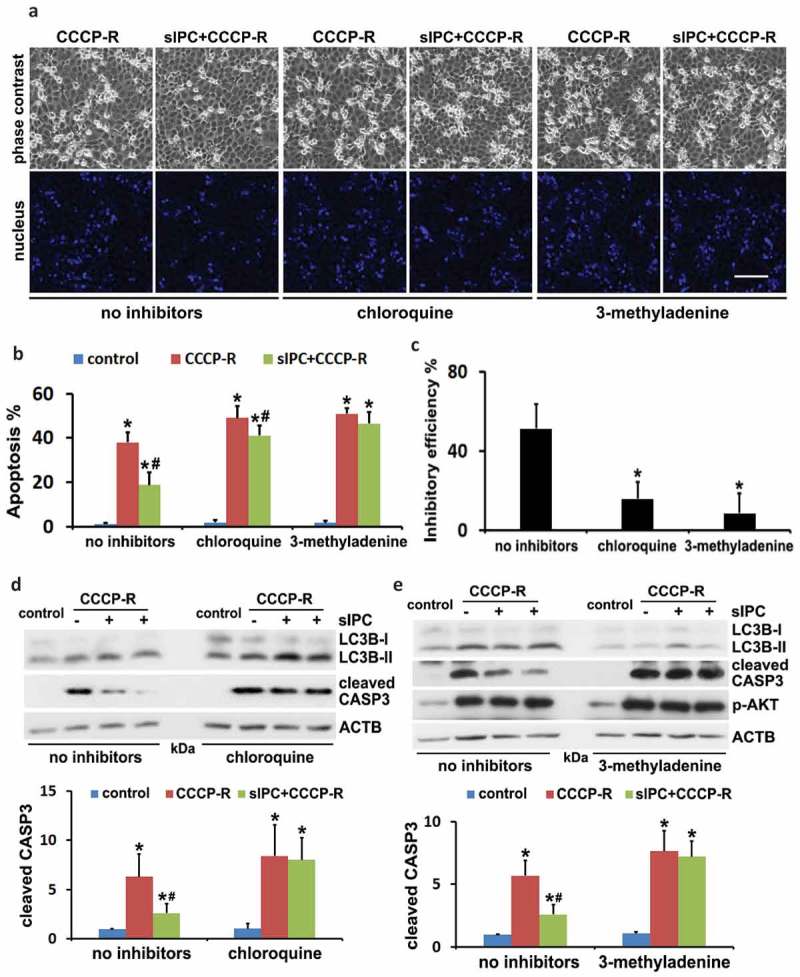
The cytoprotection of in vitro sIPC is diminished by autophagy inhibitors in RPTC cells. RPTC cells were subjected to: (1) control; (2) CCCP-R; (3) sIPC + CCCP-R in the absence or presence of chloroquine (20 μM) and 3-methyladenine (10 mM). Both inhibitors were used for 1-h pretreatment and during 2-h recovery from prolonged CCCP treatment. Cells were collected for morphological and immunoblot analyses. (a) Representative images of phase contrast and fluorescence microscopy showing cellular and nuclear morphology of apoptosis. Scale bar: 200 μm. (b) Quantification of cell apoptosis. Data are expressed as mean ± SD. *, *P* < 0.05, significantly different from the control group; #, *P* < 0.05, significantly different from CCCP-R group. (c) Analysis of apoptosis inhibitory efficiency by sIPC. Data are expressed as mean ± SD. *, *P* < 0.05, significantly different from the group without inhibitors. (d and e) Immunoblots of LC3B and cleaved CASP3. ACTB was used as a loading control. The molecular mass marker lanes were labelled as kDa. For densitometric analysis of cleaved CASP3, after normalization with ACTB, the protein signals of the control were arbitrarily set as 1, and the signals of other conditions were normalized to the control to calculate fold changes. Data are expressed as mean ± SD. *, *P* < 0.05, significantly different from the control group; #, *P* < 0.05, significantly different from CCCP-R group.

To elucidate the role of autophagy in the cytoprotection of in vitro preconditioning, we examined the effects of autophagy inhibitors, chloroquine and 3-methyladenine. The inhibitory effects of these drugs on autophagy were verified by LC3B immunoblots (Figure 7(d and e), LC3B). In the absence of autophagy inhibitors, prolonged CCCP treatment induced 38% apoptosis in RPTC cells, which was reduced to 18% by sIPC (Figure 7(a,b), CCCP-R vs sIPC + CCCP-R, no inhibitors). However, the protective effects of sIPC were largely compromised by autophagy inhibitors. As shown in Figure 7(a,b), in the presence of chloroquine or 3-methyladenine, sIPC only slightly suppressed tubular cell apoptosis (CCCP-R vs sIPC + CCCP-R, chloroquine or 3-methyladenine). By calculation, sIPC had 51% inhibitory efficiency on apoptosis in the cells with intact autophagy, which was reduced to 16% by chloroquine and to 9% by 3-methyladenine, respectively (Figure 7(c)). Both chloroquine and 3-methyladenine sensitized RPTC cells to prolonged CCCP-induced apoptosis (Figure 7(a,b), CCCP-R, no inhibitors vs CCCP-R, chloroquine or 3-methyladenine). Consistent with the morphological observations, sIPC significantly suppressed caspase activation in prolonged CCCP-treated cells, but this effect disappeared when the inhibitors impaired autophagy (Figure 7(d,e), cleaved CASP3). Together, these results confirm the involvement of autophagy in the sIPC protection in vitro during CCCP treatment of renal tubular cells.

### In vitro sIPC promotes mitophagy in RPTC cells during prolonged CCCP treatment

To further investigate the mechanism that contributes to the cytoprotection of sIPC, we performed a comprehensive analysis of mitophagy at various stages (initiation, progression, and degradation) in the in vitro model. First, RPTC cells were transfected with a plasmid encoding GFP-LC3 and incubated with MitoTracker Red CMXRos to label autophagosomes and mitochondria, respectively. The cells were then subjected to sIPC and prolonged CCCP treatment in the presence of chloroquine to block autolysosomal degradation. After treatment, we measured colocalization of mitochondria with autophagosomes by fluorescence microscopy. Consistent with our previous work [36], mitochondria in control RPTC cells were filamentous with a tubular or thread-like appearance and often interconnected to form a network (Figure S9(a), control, MitoTracker Red). A few GFP-LC3 puncta were detected, indicating a basal level of autophagy in control cells (Figure S9(a), control, GFP-LC3). However, GFP-LC3 puncta containing mitochondria were rarely seen in RPTC cells under control condition (Figure S9(a), control, overlap). sIPC alone enhanced the formation of GFP-LC3 puncta but only a very few were co-stained with mitochondria (Figure S9(a), sIPC). Following prolonged CCCP treatment, numerous GFP-LC3 puncta appeared in RPTC cells (Figure S9(a), CCCP-R, GFP-LC3). Meanwhile, mitochondria became fragmented into short rods or spheres and the mitochondrial network broke down (Figure S9(a), CCCP-R, MitoTracker Red). Notably, a portion of these fragmented mitochondria were colocalized with GFP-LC3 puncta as shown in the enlarged overlapping image, indicating the formation of mitophagosomes (Figure S9(b), CCCP-R). sIPC further promoted this colocalization, suggesting an increased autophagic sequestration of damaged mitochondria by preconditioning (Figure S9(b), sIPC + CCCP-R). We then performed quantitative analysis. Consistent with the data in Figure S8, sIPC did not alter the induction of general autophagy during prolonged CCCP treatment, as indicated by similar amount of total GFP-LC3 puncta (autophagosomes) (Figure S9(c)). Nevertheless, the number of colocalizing GFP-LC3 puncta per cell (mitophagosomes) was significantly increased from 16 in CCCP-R group to 29 in sIPC + CCCP-R group (Figure S9(d)). Accordingly, the percentage of colocalizing GFP-LC3 puncta was also increased from 19% to 35% by sIPC (Figure S9(e)). Together, these results demonstrate that sIPC enhances the initiation of mitophagy (early mitophagy) in RPTC cells during subsequent CCCP treatment.

To visualize the delivery of mitophagosomes to hydrolase-containing lysosomes, we transfected RPTC cells with COX8-EGFP-mCherry, a tandem fluorescent-tagged mitochondrial targeting sequence of inner membrane protein COX8 (cytochrome c oxidase subunit 8). This approach is based on the same principle validated in mito-QC transgenic mice and has been effective for monitoring mitophagy flux in different types of cultured cells [37,38]. Control RPTC cells showed a yellow staining of filamentous mitochondria with the merge of both green (EGFP) and red (mCherry) signals (Figure S10(a), control). In contrast, mitochondria in prolonged CCCP-treated cells became fragmented and distinct red-only puncta appeared, indicating the formation of mitolysosomes (Figure S10(a), CCCP-R). Importantly, more red-only puncta were induced by sIPC, suggesting a higher mitochondrial turnover under this condition (Figure S10(a), sIPC + CCCP-R). The delivery of fragmented mitochondria to lysosomes was completely abolished by chloroquine, further supporting the role of autophagy in mediating mitochondria turnover in our in vitro model (Figure S10(a), CQ + sIPC + CCCP-R). Quantitatively, prolonged CCCP treatment led to an average of 9 mitolysosomes per cell, which was markedly increased to 23 by sIPC (Figure S10(b)). Together, these results indicate that sIPC also promotes the progression of mitophagy from mitophagosomes to mitolysosomes (late mitophagy) in RPTC cells during subsequent CCCP treatment.

Mitochondrial degradation and clearance within the lysosome represents the completion of mitophagy flux. To monitor this final step, we stained RPTC cells with MitoTracker Red CMXRos and measured the amount of remaining mitochondria after CCCP treatment. Control RPTC cells displayed an intact filamentous mitochondrial network, with 17% of cellular area positively stained with the mitochondrial marker (Figure S11(a and b), control). Prolonged CCCP induced mitochondrial fragmentation and reduced the mitochondrial mass to 12% of cellular area (Figure S11(a and b), CCCP-R). Pretreatment with sIPC enhanced the clearance of damaged mitochondria, leading to a further decrease of MitoTracker Red staining to 9.7% of cellular area (Figure S11(a and b), sIPC + CCCP-R). To verify whether this mitochondrial degradation is autophagy-dependent, we examined the effect of chloroquine. Prolonged CCCP-induced degradation of mitochondria was partially suppressed by chloroquine, and more importantly, the promoting effects of sIPC on mitochondrial clearance was also abolished (Figure S11(a and b), + CQ). Together, these results suggest that autophagy indeed contributes to the clearance of impaired mitochondria and sIPC increases mitophagy degradation and flux in RPTC cells during prolonged CCCP treatment.

### In vitro sIPC improves mitochondrial function and reduces ROS generation in prolonged CCCP-treated RPTC cells

**Figure 8.**
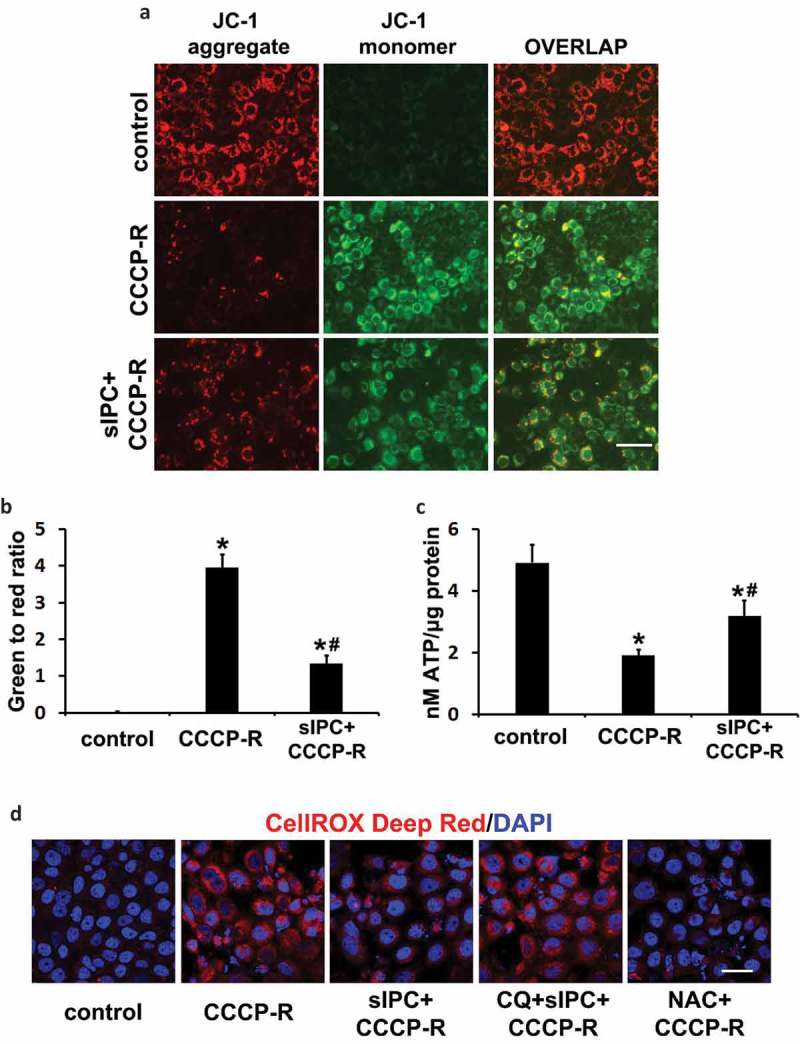
In vitro sIPC suppresses mitochondrial depolarization, improves ATP production and reduces ROS generation in prolonged CCCP-treated RPTC cells. RPTC cells were incubated with 1 μg/ml JC-1 for 1 h and then treated with: (1) control; (2) CCCP-R; (3) sIPC + CCCP-R. Live cells were collected 1 h after reperfusion for fluorescence microscopy. (a) Representative images of JC-1 staining showing red fluorescence of JC-1 aggregate and green signal of monomer. Scale bar: 50 μm. (b) Quantification of the ratio of green to red fluorescence. (c) RPTC cells were subjected to: (1) control; (2) CCCP-R; (3) sIPC + CCCP-R. Thirty min after reperfusion cells were collected for quantitative determination of ATP by a luciferin-luciferase luminescence assay. Data in (b and c) are expressed as mean ± SD. *, *P* < 0.05, significantly different from the control group; #, *P* < 0.05, significantly different from CCCP-R group. (d) RPTC cells were subjected to: (1) control; (2) CCCP-R; (3) sIPC + CCCP-R; (4) CQ + sIPC + CCCP-R; (5) NAC + CCCP-R. After treatment cells were incubated with 5 μM CellROX Deep Red reagent for 30 min. ROS generation was visualized by fluorescence microscopy. Scale bar: 50 μm.

To determine whether an increased clearance of damaged mitochondria by sIPC-mediated mitophagy would improve mitochondrial function, we first analyzed the effect on mitochondrial membrane potential in CCCP-treated RPTC cells using 5,5ʹ,6,6ʹ-tetrachloro-1,1ʹ,3,3ʹ-tetraethylbenzimidazolylcarbocyanine iodide (JC-1). JC-1 is a dichromatic dye that exhibits potential-dependent accumulation in mitochondria. It exists as either a green-fluorescent monomer at depolarizing mitochondria or a red-fluorescent aggregate at polarizing mitochondria, thus allowing for the assessment of mitochondrial polarization states. The ratio of green to red fluorescence depends only on the mitochondrial membrane potential and not on other factors such as mitochondrial size, shape and density that may influence single-component fluorescence signals [39]. In control RPTC cells, mitochondria were marked by punctate red fluorescence of JC-1 with green signal barely detected, indicating good mitochondrial membrane potential (Figure 8(a), control). In sharp contrast, prolonged CCCP treatment led to a drastic decrease of red JC-1 fluorescence in RPTC cells; instead, many cells displayed intense, mostly-diffuse green monomer fluorescence (Figure 8(a), CCCP-R). The shift from red to green JC-1 fluorescence indicated the loss of mitochondrial membrane potential in these cells. Of interest, sIPC significantly suppressed mitochondrial depolarization during prolonged CCCP treatment. The amount of punctate red JC-1 fluorescence was increased by sIPC, which was accompanied by a decrease of green monomer JC-1 fluorescence in both the amount and the intensity (Figure 8(a), sIPC + CCCP-R). Quantitative analysis further verified the morphological observations. The ratio of green to red fluorescence in control cells was 0.03. Following prolonged CCCP treatment, this ratio was induced to 3.9 and sIPC remarkably reduced it to 1.3 (Figure 8(b)). Mitochondrial membrane potential is critical for maintaining the physiological function of the respiratory chain to generate ATP. We then examined the effects of sIPC on ATP production. Control RPTC cells had 4.9 nM ATP/µg protein on average, which was reduced to 1.9 in prolonged CCCP-treated cells. sIPC partially yet significantly improved the capacity of the cells to generate energy and under this condition, the amount of ATP was increased to 3.2 nM/µg protein (Figure 8(c)). Together, these results suggest that sIPC, via enhancing autophagic clearance of damaged mitochondria, plays an important role in mitochondrial quality control in prolonged CCCP-treated RPTC cells.

ROS generated by damaged mitochondria contributes significantly to oxidative stress, which plays an important role in IRI in many tissues including the kidney. To further determine the association of mitophagy with the renoprotection of sIPC, we measured ROS generation in RPTC cells using the fluorogenic probe, CellROX Deep Red. As shown in Figure 8(d), control cells were negative of the staining as this cell-permeable dye is non-fluorescent in a reduced state. Following prolonged CCCP treatment, intense red fluorescence appeared, indicating ROS production. Remarkably, sIPC reduced ROS generation, and this beneficial effect was compromised in the presence of chloroquine. The specificity of this reagent in detecting oxidative stress was further confirmed by the inhibitory effects of N-acetylcysteine (NAC), an anti-oxidant compound (Figure 8(d)). Together with the data presented above, these results suggest that, by promoting mitophagy, sIPC attenuates the generation of ROS and prevents tubular cell apoptosis during subsequent CCCP treatment.

### Knockdown of Pink1 impairs mitophagy flux and eliminates sIPC-mediated cytoprotection in prolonged CCCP-treated RPTC cells

**Figure 9.**
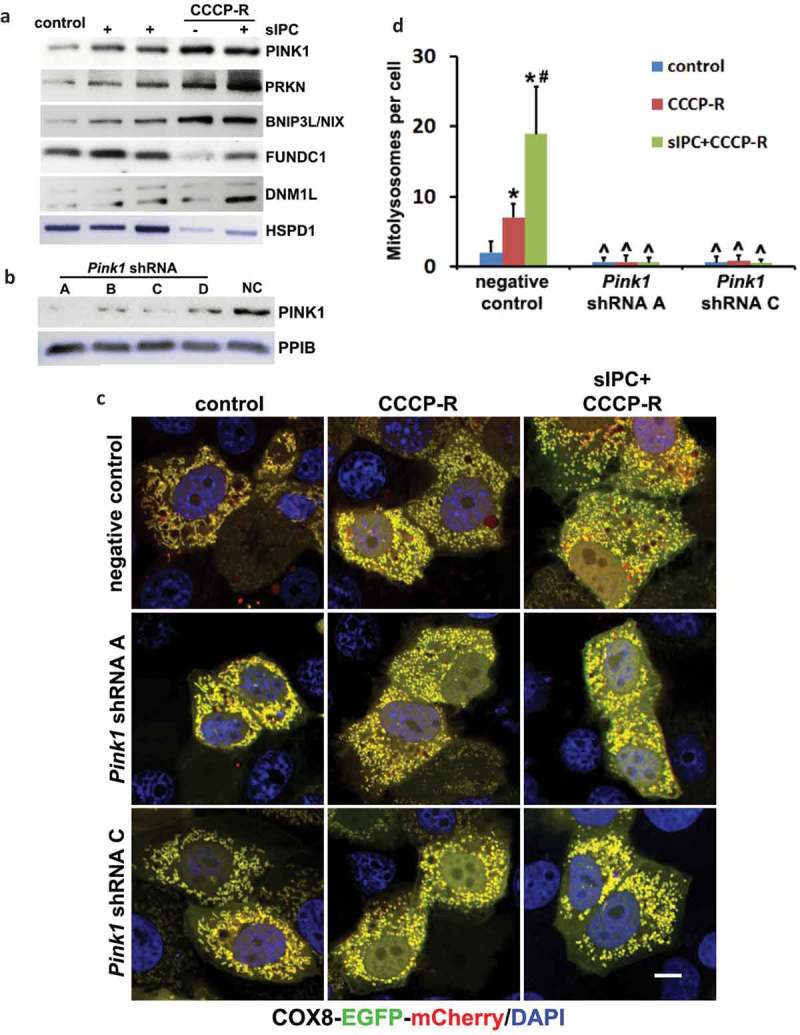
Knockdown of *Pink1* inhibits mitophagy flux in CCCP-treated RPTC cells. (a) RPTC cells were subjected to: (1) control; (2) sIPC; (3) CCCP-R; (4) sIPC + CCCP-R. After treatment mitochondrial fractions were collected for immunoblot analysis of multiple mitophagy-related proteins including PINK1, PRKN, BNIP3L/NIX, FUNDC1 and DNM1L. HSPD1, a mitochondrial matrix protein, was used as a loading control. (b) RPTC cells were infected with retroviral *Pink1* shRNA constructs (A–D) and a negative control (NC) construct. Upon puromycin selection, stable cells were collected for immunoblot analysis of PINK1. PPIB was used as a loading control. Based on the inhibitory effects, the stable cells (negative control, *Pink1* shRNA A, *Pink1* shRNA C) were transfected with COX8-EGFP-mCherry and then treated with: (1) control; (2) CCCP-R; (3) sIPC + CCCP-R. Cells were collected for fluorescence microscopy. (c) Representative images of mitolysosome formation. Scale bar: 10 μm. (d) Quantitative analysis of the number of mitolysosomes per cell. Data are expressed as mean ± SD. *, *P* < 0.05, significantly different from the control group; #, *P* < 0.05, significantly different from CCCP-R group; ^, *P* < 0.05, significantly different from the corresponding groups in negative control cells.

As shown in Figure 5, renal IRI activated PINK1 in kidneys, which was accompanied by the degradation of multiple mitochondrial proteins and more importantly, the induction of mitophagy flux in proximal tubules. To gain some insights into the regulation of mitophagy in our experimental models, we examined the expression of several mitophagy-related proteins in the mitochondrial fractions of RPTC cells. sIPC-only treatment induced a moderate PINK1 accumulation in mitochondrial fractions. Following prolonged CCCP treatment, the mitochondrial accumulation of PINK1 was further enhanced (Figure 9(a), PINK1). Consistently, PRKN, an E3-ubiquitin ligase that can be recruited onto the surface of damaged mitochondria by activated PINK1 to initiate mitophagy, was also accumulated in mitochondria at a moderate level in response to sIPC-only and further increased after prolonged CCCP treatment (Figure 9(a), PRKN). In addition to these two regulators, BNIP3L/NIX (BCL2 interacting protein 3 like), a mitophagy receptor protein, was also induced and accumulated in mitochondria under both sIPC and prolonged CCCP conditions (Figure 9(a), BNIP3L/NIX). Similar changes were also shown in DNM1L (dynamin 1 like), a well-known mitochondrial fission regulator (Figure 9(a), DNM1L). In contrast, the mitochondrial expression of FUNDC1 (FUN14 domain containing 1), another receptor protein that has been shown to induce mitophagy under hypoxia conditions, was not altered by sIPC alone but significantly reduced during prolonged CCCP treatment (Figure 9(a), FUNDC1). These results suggest a possibility that multiple signaling pathways might contribute to the regulation of sIPC-related mitophagy in our experimental models.

To further determine the role of PINK1-PRKN pathway, we infected RPTC cells with retroviral *Pink1* shRNA constructs and selected stable cells with puromycin. The efficiency of gene silencing was verified by immunoblot analysis of PINK1. Compared with negative control vector, the expression of PINK1 was suppressed by all 4 *Pink1* shRNAs in the stable cells, with A and C more effective than B and D (Figure 9(b)). We therefore chose the cells infected with negative control, *Pink1* shRNA A, and *Pink1* shRNA C for further analysis. These cells were transiently transfected with COX8-EGFP-mCherry and the delivery of mitophagosomes to lysosomes to form mitolysosomes was examined. In negative control transfection cells, the formation of mitolysosomes was detected at a very low basal level under control condition and mitochondria formed a yellow-stained network (Figure 9(c), negative control, control). Following prolonged CCCP treatment, mitochondria became fragmented and distinct red-only puncta appeared, indicating the formation of mitolysosomes (Figure 9(c), negative control, CCCP-R). More red-only puncta were induced by sIPC in prolonged CCCP-treated cells, suggesting an enhanced mitophagy flux (Figure 9(c), negative control, sIPC + CCCP-R). Quantitatively, an average of 7 mitolysosomes per cell was induced during prolonged CCCP treatment, which was markedly increased to 19 by sIPC (Figure 9(d), negative control). In contrast, the formation of mitolysosomes was almost completely blocked in *Pink1* shRNA knockdown cells under all 3 treatment conditions (Figure 9(c,d), *Pink1* shRNA A, *Pink1* shRNA C). These results suggest that PINK1-PRKN pathway plays a central role in mitophagy initiation and progression in this experimental condition.

**Figure 10.**
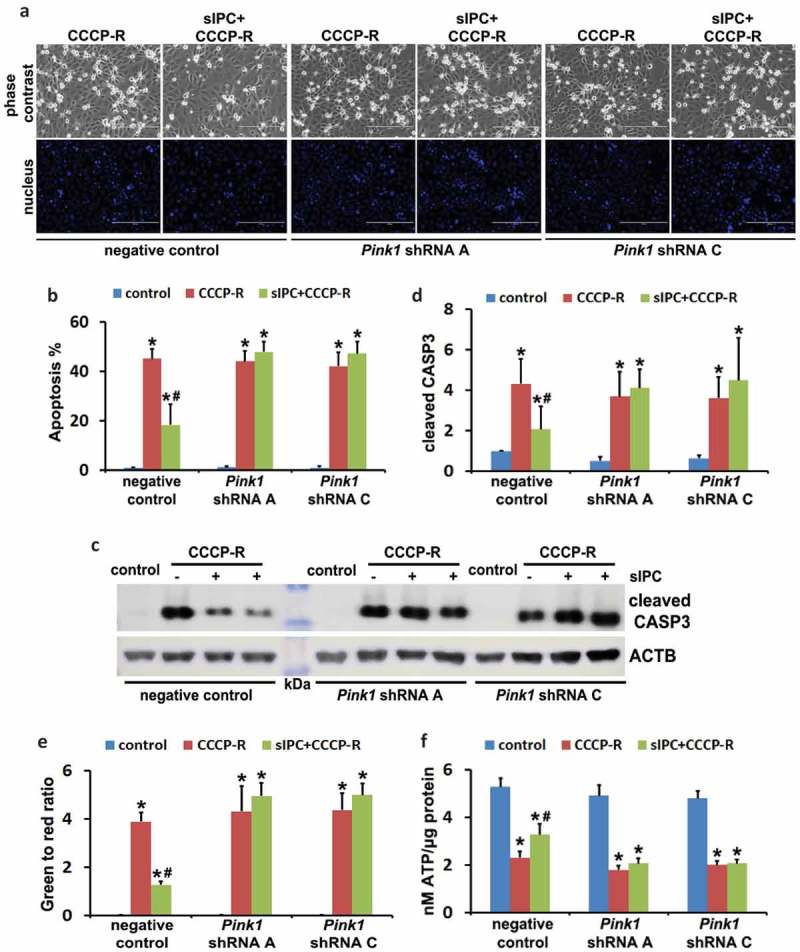
In vitro sIPC-mediated cytoprotection is compromised by *Pink1* knockdown in RPTC cells. RPTC stable cell lines (negative control, *Pink1* shRNA A, *Pink1* shRNA C) were established as described in Figure 9. The cells were then treated with: (1) control; (2) CCCP-R; (3) sIPC + CCCP-R. After treatment cells were collected for morphological, biochemical and immunoblot analyses. (a) Representative images of phase contrast and fluorescence microscopy showing cellular and nuclear morphology of apoptosis. Scale bar: 200 μm. (b) Quantification of cell apoptosis. (c) Representative images of cleaved CASP3 immunoblot. ACTB was used as a loading control. The molecular mass marker lane was labelled as kDa. (d) Densitometric analysis of cleaved CASP3 immunoblots. After normalization with ACTB, the protein signals of the control in negative control cells were arbitrarily set as 1, and the signals of other conditions were normalized to calculate fold changes. (e) Quantitative analysis of JC-1 staining (the ratio of green to red fluorescence). Cells were loaded with 1 μg/ml JC-1 for 1 h prior to CCCP treatment. Live cells were collected for fluorescence microscopy. (f) Bioluminescence assay of ATP production. Data in (b,d–f) are expressed as mean ± SD. *, *P* < 0.05, significantly different from the control group; #, *P* < 0.05, significantly different from CCCP-R group.

We next examined the effects of mitophagy inhibition on sIPC-mediated cytoprotection in RPTC cells. In negative control transfection cells, prolonged CCCP treatment induced 45% apoptosis, which was reduced to 18% by sIPC (Figure 10(a,b), negative control). Of note, the protective effects of sIPC were completely eliminated by mitophagy inhibition. In *Pink1* shRNA A cells, 44% apoptosis was induced in response to prolonged CCCP treatment. sIPC did not afford any cytoprotection (Figure 10(a,b), *Pink1* shRNA A). The loss of sIPC-mediated beneficial effects was also shown in *Pink1* shRNA C cells (Figure 10(a,b), *Pink1* shRNA C). Consistently, without mitophagy inhibition sIPC significantly suppressed caspase activation in prolonged CCCP-treated cells (Figure 10(c,d), negative control). However, when mitophagy was impaired by *Pink1* knockdown, the inhibitory effect of sIPC on caspase activation dissappeared (Figure 10(c,d), *Pink1* shRNA A, *Pink1* shRNA C). In addition, sIPC-mediated mitochondrial protection was also compromised in these mitophagy-deficient cells. As shown in Figure 10(e,f), in negative control cells, sIPC prevented the loss of mitochondrial membrane potential and promoted ATP production during prolonged CCCP treatment. These beneficial effects of sIPC were largely lost in *Pink1* knockdown cells. Together, these results provide direct evidence that mitophagy specifically contributes to the cytoprotective effect of sIPC during CCCP treatment of renal tubular cells.

## Discussion

Both IPC and autophagy/mitophagy protect against kidney injury by renal ischemia-reperfusion. However, the connection between these two in the kidney has not been reported. Using both in vivo and in vitro models, the current study has provided the first evidence for a critical role of autophagy/mitophagy in the protective effect of renal IPC. In this study, IPC induced autophagy both in mouse proximal tubules and in cultured renal tubular cells. Inhibition of autophagy by pharmacological inhibitors (chloroquine and 3-methyladenine) or *Atg7* deletion specifically from proximal tubules eliminated the renoprotective effect of IPC. Notably, IPC did not affect the induction of general autophagy during subsequent renal IRI, but it significantly enhanced mitophagy in proximal tubular cells. Increased clearance of damaged mitochondria via mitophagy prevented mitochondrial function loss, attenuated ROS generation, and alleviated tubular cell apoptosis and kidney injury. *Pink1* knockdown in proximal tubular cells blocked mitophagy, suggesting a role of the PINK1-PRKN pathway in IPC-mediated mitophagy. Specific inhibition of mitophagy also abolished the beneficial effects of IPC. Moreover, pharmacological preconditioning with Tat-BECN1 activated autophagy/mitophagy and protected kidneys against renal IRI, further suggesting potential therapeutic applications of autophagy/mitophagy in renal IPC.

In this study, a short period of ischemia (15 min) induced autophagy in kidney proximal tubules within 1 h of reperfusion (Figure 2). Compared to a prolonged renal ischemia (28 to 30 min) that induces autophagy after 6 h of reperfusion [22], autophagy activation during renal IPC was apparently much earlier and faster. A rapid induction of autophagy within minutes in response to sub-lethal ischemia has also been shown in the heart and liver [9,10,15]. Consistently, inhibition of autophagy abolishes the protective effects of IPC in all the three organs, suggesting that autophagy may be a common adaptive mechanism that is essential for the resistance of preconditioned tissues to subsequent ischemic injury. In our experimental models, renal IPC-induced autophagy suppressed tubular cell apoptosis but not necrosis, resulting in a significant but incomplete renoprotection. These findings are different from the inhibitory effects of IPC-induced autophagy on both cell apoptosis and necrosis in the heart and liver [9,10,15], suggesting that the extent of the protection mediated by autophagy during IPC may depend on the tissue or cell type and injury duration.

Another interesting characteristic of renal IPC-induced autophagy is the unique subcellular localization of autophagic vacuoles in proximal tubular cells. Immunohistochemical staining showed that LC3B-positive granules accumulated along the brush boarder of preconditioned proximal tubules (Figure 2(c)). EM further confirmed that autophagic vacuoles induced by renal IPC were mainly seen at the apical cytoplasm of proximal tubules underneath the brush boarder (Figure 2(e), circled areas). We also observed a remarkable decrease in the number of reabsorption vacuoles in this area as compared to sham control (Figure S1). To our knowledge, these observations have not been reported previously. Normally the cytoplasm immediately beneath the brush border is well controlled for cell organelle distribution. Reabsorption vacuoles (also called secondary lysosomes) contained in this area are involved in reabsorption and degradation of small amounts of protein that have leaked through the glomerular filter. The mechanisms that contribute to the specific subcellular distribution of autophagic vacuoles during renal IPC needs to be further investigated. One possibility is that, upon induction in IPC, autophagy mainly involves the fusion of autophagosomes with reabsorption vacuoles or secondary lysosomes in IPC.

Unlike the observations in the heart and brain [9,11], in our experiment models, IPC did not change the induction of general autophagy during prolonged ischemia either in kidneys (Figure S2) or in renal tubular cells (Figure S8). However, renal IPC significantly enhanced selective mitophagy under these conditions. We first used the mito-QC transgenic mouse model to monitor mitophagy in vivo [34]. These mice carry a tandem mitochondria-targeting fluorescence probe, mCherry-GFP-FIS1, and the differential pH sensitivities between mCherry and GFP allow us to measure the delivery of mitochondria to lysosomes to form mitolysosomes in the kidney. Interestingly, in sham control mice, mitophagy occurred at a high basal level in some cortical proximal tubules, with mitolysosomes positioned predominantly at the apical side of proximal tubules toward the lumen (Figure 5(b) and S5(b)). It is unclear what causes this polarized distribution. However, given the consideration that the apical side of renal proximal tubules are enriched for a large number of ATP-dependent transporters for both excretion and reabsorption, it is not surprising that a high rate of constant mitochondrial turnover occurs in this area. In contrast to some highly mitophagic proximal tubules in renal cortex, the proximal tubules in S3 segment at the outer medulla showed minimal mitochondrial turnover under basal condition and the occurrence of mitophagy in glomeruli was rare as well (Figure S5). Mitophagy was significantly induced in proximal tubules following renal IRI, and more importantly, renal IPC further enhanced the intensity of mitolysosome formation in renal IRI (Figure 5(b,c)). These in vivo results were further verified in cultured proximal tubular cells by a comprehensive analysis of mitophagy dynamics at different stages. In the cells subjected to ATP-depletion stress, sIPC promoted the colocalization of mitochondria with autophagosomes (initiation) (Figure S9), enhanced the fusion of mitophagosomes with lysosomes to form mitolysosomes (progression) (Figure S10), and increased the degradation of mitochondrial mass (clearance) (Figure S11). These results demonstrate, unequivocally, that renal IPC enhances mitophagy in proximal tubular cells during renal IRI. Moreover, IPC-induced clearance of damaged mitochondria suppressed mitochondrial depolarization, improved ATP production and also reduced ROS generation (Figure 8), further suggesting an important role of IPC-mediated mitophagy in mitochondrial quality control and IPC renoprotection. In support of our findings, previous studies show that IPC in kidneys increases the percentage of functionally active mitochondria, prevents nitric oxide synthesis, and reduces the severity of kidney dysfunction [40]. Moreover, IPC protection is significantly less in aged animals, which is associated with impaired autophagy/mitophagy and the accumulation of dysfunctional mitochondria [41].

The upstream signaling involved in mitophagy induction by renal IPC has yet to be determined. In IPC-mediated cardioprotection, mitophagy mediated by PRKN and SQSTM1 is suggested to eliminate damaged mitochondria [21]. In our study, activation of PINK1, an important mitophagy mediator that recruits PRKN to damaged mitochondria, was also seen in kidneys following renal IPC and IRI (Figure 5(a)), which raises the possibility that PINK1-PRKN signaling pathway may also regulate mitophagy induced by renal IPC. Indeed, in cultured proximal tubular cells, both PINK1 and PRKN were induced and accumulated in mitochondria in response to IPC and prolonged CCCP treatment (Figure 9(a), PINK1, PRKN). Knockdown of *Pink1* in these cells remarkably abolished mitolysosome formation and mitophagy flux (Figure 9(c,d)), suggesting a regulatory role of the PINK1-PRKN pathway in in vitro IPC-mediated mitophagy. In addition, HIF1A (hypoxia inducible factor 1 subunit alpha) is an important mediator of IPC in the kidney. Under hypoxia conditions, both BNIP3 and BNIP3L/NIX are activated by HIF1A [5]. In cultured MEF cells, induction of mitophagy during hypoxia is regulated by HIF1A-dependent activation of BNIP3 [42]. The role of BNIP3L/NIX in inducing general autophagy and in priming damaged mitochondria for selective autophagic recognition has also been shown in CCCP-induced mitochondrial depolarization in MEF cells [43]. Consistently in our study, an accumulation of BNIP3L/NIX in mitochondria was also detected in cultured renal proximal tubular cells under both IPC and prolonged CCCP treatment conditions (Figure 9(a), BNIP3L/NIX). Whether HIF1A-BNIP3 or BNIP3L/NIX pathway contribute to mitophagy induction by renal IPC during renal IRI needs to be elucidated. Moreover, in our in vivo models, both LC3B immunohistochemical staining and EM revealed a unique subcellular distribution of autophagic vacuoles in preconditioned proximal tubules, which also happened to be at the apical side where mitophagy was induced during subsequent renal IRI (Figure 2(c,e)). Whether they are functionally connected (for instance, autophagy induced during preconditioning phase may activate certain signaling to facilitate mitophagy during subsequent injury phase) is worth further investigations.

It remains unclear as to how general autophagy is activated by renal IPC. Earlier studies in the heart show that IPC enhances the expression of a pro-survival protein, BAG1 (BCL2-associated athanogene 1), which interacts with LC3 and induces autophagy for cardioprotection [9,44]. A recent study further suggests that during cardiac IPC autophagy is induced via epigenetic repression of *Mtor* (mechanistic target of rapamycin kinase) by histone H3K9 dimethylation [45]. In the liver, induction of autophagy during IPC is associated with other stress adaptive signaling such as HMOX1 (heme oxygenase 1) and nitric oxide [15,16]. Furthermore, endoplasmic reticulum (ER) stress, through a chaperon protein HSPA5/GRP78 (heat shock protein 5), activates autophagy in neural cells during IPC [46]. Autophagy induced by cerebral IPC reduces excessive ER stress during fetal ischemia and protects neurons from ER stress-induced apoptosis [12,13]. These studies suggest inter-regulations between ER stress and autophagy and the significance to IPC-mediated neuroprotection. Cerebral IPC also induces autophagy via AMP-activated protein kinase (AMPK) and protects against ischemic stroke in rats [14]. Despite these findings, the regulatory mechanisms underlying the activation of autophagy by renal IPC need to be elucidated.

In conclusion, this study has demonstrated compelling evidence for a role of autophagy in the protective effect of IPC in kidneys. Particularly, mitophagy is specifically enhanced by IPC during subsequent injury, resulting in the removal of damaged mitochondria, improvement of mitochondrial function, reduction of ROS, and increase of cell survival. As a prominent mechanism of intrinsic adaption, IPC has gained continued attention and interest in both basic science research and clinical application. However, the translation of IPC to beneficial treatment for patients has been limited. A better understanding of the mechanisms by which IPC affords protection in various organs may facilitate the discovery of new therapeutic targets for clinical use.

## Materials and methods

### Cell lines, antibodies and reagents

The immortalized RPTC cell line was originally obtained from Dr. Ulrich Hopfer (Case Western Reserve University) and maintained in DMEM/F-12 medium supplemented with 10% fetal bovine serum and growth factors [47]. The retrovirus packaging cell line (293-Phoenix) was provided by Drs. Xiongjie Jin and Nahid F. Mivechi (Augusta University). The cells were cultured in DMEM medium with 10% fetal bovine serum. Primary antibodies: anti-LC3B (NB100-2220), anti-PINK1 (BC100-494) and anti-FUNDC1 (NBP1-81063) were from Novus Biologicals; anti-SQSTM1 (ab56416), anti-IMMT/MIC60 (ab110329), anti-COX4I1 (ab153709) and anti-PPIB (ab16045) were from Abcam; anti-TOMM20 (sc-11415), anti-PINK1 (sc-517353), anti-PRKN (sc-133167), anti-BNIP3L/NIX (sc-166332) and anti-HSPD1 (sc-13966) were from Santa Cruz Biotechnology; anti-CASP3 (9665) and anti-cleaved CASP3 (9664) were from Cell Signaling Technology; anti DNM1L (BD Biosciences, 611113); anti-ACTB (Sigma-Aldrich, A5316). Secondary antibodies for immunoblot analysis were from Jackson ImmunoResearch Laboratories. CCCP (C2759), chloroquine (C6628) and 3-methyladenine (M9281) were from Sigma-Aldrich. BECN1 peptide (Tat-BECN1, sequence: YGRKKRRQRRRGGTNVFNATFEIWHDGEFGT) and its control peptide (Tat-scrambled, sequence: YGRKKRRQRRRGGVGNDFFINHETTGFATEW) were synthesized by GenScript.

### In vitro model of sIPC and renal IRI in RPTC cells and examination of apoptosis

RPTC cells were plated in 35-mm dishes at a density of 1.0 × 10^6^ cells/dish to reach ~95% confluence by next day. To induce in vitro sIPC, cells were incubated with 20 μM CCCP in regular Ca^2+^ Krebs-Ringer buffer (in mM: 115 NaCl, 1 KH_2_PO_4_, 4 KCl, 1 MgSO_4_, 1.25 CaCl_2_, 25 NaHCO_3_, pH 7.4) for 30 min followed by 40 min of recovery in full culture medium. To mimic in vivo renal IRI, cells were treated with 20 μM CCCP in regular Ca^2+^ Krebs-Ringer buffer for 3 h followed by 2 h of recovery in full culture medium. To test the effects of autophagy inhibitors, RPTC cells were pretreated with 20 μM chloroquine or 10 mM 3-methyladenine for 1 h and the inhibitors were added back into the cells during 2-h recovery from prolonged CCCP treatment. Apoptosis was examined by morphology and caspase activation. Morphologically, cells were stained with 10 μg/ml Hoechst 33342 (Molecular Probes, H1399) to examine cellular and nuclear morphology by phase contrast and fluorescence microscopy, respectively. Cells with typically apoptotic characterizations were counted to estimate apoptosis percentage. To measure caspase activation, whole cell lysates were collected for immunoblot analysis of cleaved CASP3.

### Animals

C57BL/6 mice were originally purchased from Jackson Laboratory. The PT-*atg7* KO mouse model was generated and characterized in our lab [23]. The autophagy reporter mouse model, which is transgenic of a tandem RFP-GFP-LC3 fusion gene under the control of *CAG* promoter (CAGp-RFP-GFP-LC3 mice), was established recently [28]. The mitophagy reporter mouse model, mito-QC that carries a tandem mCherry-GFP tag fused with mitochondrial outer membrane protein FIS1, was generated lately [34]. Male mice 8- to 10-wk old were used for experiments. Mice were housed in a pathogen-free animal facility of Charlie Norwood VA Medical Center under a 12–12-h light-dark pattern with free access to food and water. All animal experiments were conducted in accordance with a protocol approved by the Institutional Animal Care and Use Committee of Charlie Norwood VA Medical Center.

### Experimental models of renal IPC and renal IRI in mice

Before ischemia surgery, mice were anesthetized with pentobarbital (50 mg/kg, i.p.) and kept on a Homeothermic Blanket Control Unit (Harvard Apparatus, 507220F, Holliston, MA) to monitor and maintain body temperature at ~36.5ºC. Buprenorphine (0.05 mg/kg, i.p.) was used for analgesia before and after surgery. To induce renal IPC, renal pedicles were exposed for bilateral clamping to induce 15-min ischemia. The clamps were then released for reperfusion for 1 h. Mice were then subjected to 27-min bilateral renal ischemia followed by reperfusion for up to 48 h to induce renal IRI as described in our previous work [22,36]. During surgery additional anesthesia and analgesia were given based on response to tail pinch. In some experiments, mice were subjected to renal IPC only without subsequent renal IRI and kidneys were collected 1 h after preconditioning. Sham control mice underwent the same operation without renal pedicle clamping. To test the effects of autophagy inhibitors, C57BL/6 mice were pretreated with either chloroquine (60 mg/kg, i.p.) or 3-methyladenine (30 mg/kg, i.p.) 1 d and 1 h before surgery and continuously subjected to the inhibitors daily throughout IPC and renal IRI duration. To determine the effects of autophagy-inducing peptide, C57BL/6 mice were given Tat-BECN1 or Tat-Scramble (20 mg/kg, i.p.) 4 h before 27-min renal ischemia surgery. Mice were sacrificed 48 h after renal IRI for histological and biochemical analyses.

### Examination of general autophagy in kidneys and in cells

1. Autophagy in RPTC cells and in kidney tissues was examined by immunoblot analysis of LC3B and SQSTM1 as described recently [22,23,30]. In some experiments, LC3B-II turnover assay was performed to monitor autophagic flux by comparing the amount of LC3B-II in the absence vs presence of chloroquine.

2. A polymer-based immunohistochemical staining of LC3B was also used to determine autophagosome formation in proximal tubules as described recently [30]. After perfused fixation with 4% paraformaldehyde, mice were sacrificed and kidneys were post-fixed in the same fixative overnight and routinely paraffin-embedded. Tissue sections (4 μm) were deparaffinized and incubated with 1 mM EDTA, 0.05% Tween 20 (Fisher Scientific, BP337-500), pH 8.0 at 95–100°C for antigen retrieval. After incubated with 3% H_2_O_2_ and with a blocking buffer (2% bovine serum albumin [Sigma-Aldrich, A9647], 0.2% milk, 2% normal goat serum [Jackson ImmunoResearch Laboratories, 005–000-121], 0.8% Triton X-100 [Sigma-Aldrich, T9284]), the slides were exposed to 1:500–1000 anti-LC3B at 4°C overnight and incubated with Dako EnVision+ System horse radish peroxidase-labelled polymer goat anti-rabbit secondary antibody (Dako North America, K4002) for 30 min at room temperature. Signals were visualized with a DAB Kit (Vector Laboratories, SK-4100). For quantification, 10 to 20 fields (400×) were randomly selected from each slide and the amount of LC3B dots per proximal tubule was evaluated using ImageJ (National Institutes of Health).

3. Examination of autophagic vacuoles in renal tissues by EM was described in our recent work [22,48]. Briefly, after treatment mice were sacrificed and immediately perfused with 10 ml (10 units/ml) heparin, followed by 50 ml fixative (100 mM sodium cacodylate, 2 mM CaCl_2_, 4 mM MgSO_4_, 4% paraformaldehyde, 2.5% glutaraldehyde). Kidneys were harvested and post-fixed in the same fixative. An approximate 1 mm^3^ of tissue cube was collected from each kidney, including a portion of renal cortex and outer medulla for standard EM processing. The structures of autophagic vacuoles in proximal tubular cells were revealed at high magnification (10,000×).

4. Autophagy dynamics was analyzed in RPTC cells expressing mRFP-GFP-LC3 and in CAGp-RFP-GFP-LC3 transgenic mice as described recently [30]. RPTC cells were transiently transfected with mRFP-GFP-LC3 (ptfLC3; Addgene, 21074; deposited by Dr. Tamotsu Yoshimuri) [29]. After treatment cells were briefly fixed with 4% paraformaldehyde and mounted with Prolong Diamond antifade reagent (Molecular Probes, P36962) for fluorescence microscopy (Zeiss 780 upright confocal microscope, Carl Zeiss USA, Thornwood, NY). For quantitative analysis, approximately 100 transfected cells from 10 to 20 random fields (630×) were analyzed in each condition. The numbers of yellow (colocalizing GFP-LC3 with RFP-LC3) puncta per cell and total red (RFP-LC3) puncta per cell were counted separately using ImageJ. The number of autophagosomes was indicated by yellow puncta and the number of autolysosomes was obtained by subtracting yellow puncta from total red puncta. The number of autolysosomes was further divided by the total number of RFP-LC3 puncta to indicate the autophagic flux rate. For in vivo experiments, after treatment CAGp-RFP-GFP-LC3 mice were perfused with 4% paraformaldehyde. Kidneys were further fixed overnight with the same fixative, balanced with 30% sucrose (Fisher Scientific, S5-500), and embedded in Tissue-Tek® O.C.T. compound (Sakura Finetek USA, 4583) for cryo-section (5 μm) and confocal microscopy. For each section, 8 to 10 fields (630×) were selected randomly and quantitative analysis was performed by the method described above in RPTC cells.

### Analysis of mitophagy in kidneys of mito-QC mice

Mito-QC is a newly established transgenic mouse model that expresses a tandem mCherry-GFP tag fused to the mitochondrial outer membrane protein FIS1 [34]. As described above, based on the differential sensitivities of mCherry and GFP to the acidic lysosomal environment, this reporter mouse model can accurately measure the delivery of mitophagosomes to lysosomes and mitophagy flux in multiple organs including the heart, brain, liver, spleen, skeletal muscles, and kidney [34]. For our in vivo experiments, following renal IRI mito-QC mice were perfused with 4% paraformaldehyde. Kidneys were further fixed overnight with the same fixative, balanced with 30% sucrose, and embedded in Tissue-Tek® O.C.T. compound for cryo-section (5 μm) and confocal microscopy. For each section, 8 to 10 fields (400×) from renal cortex and outer stripe of outer medulla were selected randomly and the number of mCherry-only puncta (mitolysosomes) per 400× field (glomeruli excluded) was counted for quantification using ImageJ.

### Assessment of mitophagy in RPTC cells

A variety of complementary methods were used to monitor mitophagy in RPTC cells at different stages:

1. The initiation of mitophagy was determined by the colocalization of mitochondria and autophagosomes. RPTC cells were transiently transfected with GFP-LC3 (generously provided by Dr. Tamotsu Yoshimuri) [49]. Twenty-four h after transfection, cells were incubated with 50 nM MitoTracker Red CMXRos (Molecular Probes, M7512) in full culture medium for 30 min. Following CCCP treatment in the presence of chloroquine, cells were briefly fixed in 4% paraformaldehyde and mounted with Prolong Diamond antifade reagent for confocal fluorescence microscopy. For quantification, approximately 50 transfected cells from 8 to 10 random fields (630×) were analyzed in each condition. The numbers of total GFP-LC3 puncta per cell (autophagosomes) and colocalizing GFP-LC3 puncta per cell (mitophagosomes) were counted separately using ImageJ. The percentage of colocalizing GFP-LC3 puncta in total GFP-LC3 puncta was also calculated. It is noteworthy that MitoTracker Red CMXRos carries a thiol-reactive chloromethyl group that covalently binds to the reduced thiols present in mitochondrial matrix protein. For this reason, this dye can remain in the mitochondria regardless of altered mitochondrial function or mitochondrial membrane potential after CCCP treatment and is also retained after fixation.
2. The progression of mitophagy was determined by the delivery of mitophagosomes to lysosomes. RPTC cells were transiently transfected with COX8-EGFP-mCherry (Addgene, 78520; deposited by Dr. David Chan) for 24 h [37]. After CCCP treatment, cells were briefly fixed in 4% paraformaldehyde and mounted with Prolong Diamond antifade reagent for confocal fluorescence microscopy. For quantification, approximately 80 to 100 transfected cells from 10 to 20 random fields (630×) were analyzed in each condition and the number of mCherry-only puncta (mitolysosomes) per cell was counted using ImageJ.
3. The clearance of mitochondria was determined by the loss of mitochondrial mass. RPTC cells were incubated with 50 nM MitoTracker Red CMXRos in full culture medium for 30 min. Following CCCP treatment cells were briefly fixed in 4% paraformaldehyde and mounted with Prolong Diamond antifade reagent for confocal fluorescence microscopy. To verify the role of autophagy in mediating mitochondrial degradation, chloroquine was used to block autolysosomal acidification. For quantification, 15 to 20 fields (630×) were randomly selected in each condition and the intensity and area of MitoTracker Red staining was analyzed using ImageJ.

### Assessment of mitochondrial membrane potential, ATP production and ROS generation in RPTC cells

1. Mitochondrial membrane potential was examined in live cells by the mitochondrial membrane potential sensitive dye JC-1. RPTC cells were incubated with 1 µg/ml JC-1 (Molecular Probes, T3168) in full culture medium for 1 h. After treatment, live cells were directly examined by fluorescence microscopy (Zeiss 780 inverted confocal microscope, Carl Zeiss USA, Thornwood, NY). For quantitative analysis, 8 to 10 fields (200×) were randomly selected in each condition. The shift of JC-1 fluorescence from red to green was measured and the ratio of green to red fluorescence was analyzed using ImageJ.

2. ATP production was measured by a luciferin-luciferase bioluminescence assay. After treatment RPTC cells were extracted with 1x Reporter lysis buffer (Promega, E397A). The cells were collected and then subjected to a single freeze-thaw cycle (freeze at −80ºC for 5 min and thaw at room temperature for 10 to 15 min) to ensure complete lysis. The cell lysates were vortexed for 10 to 15 s and then centrifuged at 12,000 g for 15 s at room temperature. The supernatants were collected for ATP measurement using an ATP determination kit (Molecular Probes, A22066) as described by the manufacturer. For each measurement, a standard curve was constructed using ATP standard solutions. The amount of ATP was calculated from the standard curve and normalized with protein concentration (nM ATP/µg protein).

3. ROS generation was also measured to monitor mitochondrial function. Following CCCP treatment, RPTC cells were incubated with 5 μM CellROX Deep Red reagent (Molecular Probes, C10422) for 30 min. After brief fixation in 4% paraformaldehyde, cells were mounted with Prolong Diamond antifade reagent and ROS generation was visualized by fluorescence microscopy.

### Mitochondrial fractionation

After treatment RPTC cells were scraped and harvested with homogenization buffer (10 mM HEPES-NaOH, pH 7.5, 220 mM mannitol, 70 mM sucrose) on ice. The cells were homogenized with 10 strokes using a syringe with 27-gauge needle and centrifuged at 500 g for 5 min at 4ºC. The supernatants were collected and further centrifuged at 8,000 g for 5 min at 4ºC. The pellets containing crude mitochondria were washed with homogenization buffer and dissolved in 2% SDS buffer described below for immunoblot analysis of various mitophagy-related proteins.

## *Generation of stable RPTC cells by retroviral* Pink1 *shRNA infection*

*Pink1* rat shRNA (TR705269A, B, C, D) and its negative control plasmids were purchased from OriGene Technologies. 293-Phoenix cells were transiently transfected with *Pink1* shRNA and its negative control constructs using Lipofectamine 2000 (Life Technologies, 11668). Forty-eight h after transfection, the culture media containing a high titer of viruses were collected and filtered to remove cell debris. To establish stable cells, RPTC cells were seeded in 35-mm dishes at a density of 0.5 × 10^6^ cells/dish to reach ~50% confluence by next day. The viral stocks and 2 µg/ml polybene (Santa Cruz Biotechnology, sc-134220) were added into RPTC culture medium for infection. Forty-eight h after infection, the cells were selected with 2.5 µg/ml puromycin (TaKaRa Bio USA, 631305) for 10 d and then collected to examine the efficiency of gene silencing by immunoblot analysis of PINK1.

### Renal function, histology and TUNEL assay

Renal function was determined by BUN and serum creatinine using commercial kits (Stanbio Laboratory, 0580 and 0420). In brief, blood samples were collected for coagulation and centrifugation at room temperature to collect serum. For BUN, the reaction was conducted at 100°C for 12 min and the absorbance at 520 nm was recorded by the end of reaction. For serum creatinine, samples were added to a pre-warmed (37°C) reaction mixture and the absorbance at 510 nm was monitored kinetically at 20 and 80 s of reaction. BUN and creatinine levels (mg/dl) were then calculated based on standard curves. Kidney tissues were fixed with 4% paraformaldehyde, embedded in paraffin, and sectioned at 4 μm. H-E staining was performed using standard procedures. As described before [22,23], renal tubules with the following histopathological changes were considered injured: loss of brush border, tubular dilation and disruption, cast formation, cell lysis and with sloughed debris in tubular lumen. Tissue damage was examined in a blind manner and scored by the percentage of damaged tubules: 0, no damage; 1, <25%; 2, 25–50%; 3, 51–75%; 4, >75%. Apoptotic cells were identified by TUNEL assay using an in situ cell death detection kit (Roche Applied Science, 12156792910) as described previously [22,23]. Briefly, tissue sections were deparaffinized and permeabilized with 0.1 M sodium citrate, pH 6.0 at 65°C for 30 min. The sections were then incubated with a TUNEL reaction mixture for 1 h at 37ºC in a humidified, dark chamber. Positive nuclear staining was detected by fluorescence microscopy. For quantification, 10 to 20 fields (200×) were randomly selected from each tissue section and the amount of TUNEL-positive cells per mm^2^ tissue was evaluated.

### Immunoblot analysis

RPTC cells and kidney tissues from cortex and outer medulla were lysed in 2% SDS buffer (62.5 mM Tris-HCl, pH 6.8, 2% SDS, 10% glycerol) containing protease inhibitor cocktail (Sigma-Aldrich, P8340) and Benzonase nuclease (EMD Millipore, 70746). Protein concentration was determined by Pierce BCA protein assay kit (Thermo Scientific, 23225). Equal amounts (10 to 20 μg for cell lysate, 50 to 100 μg for tissue lysate) of protein were loaded in each lane for electrophoresis and immunoblot analysis using standard methods.

### Statistics

Qualitative data including immunoblots and images are representatives of at least 3 experiments. Quantitative data were expressed as means ± SD (standard deviation). Statistical analysis was conducted using the GraphPad Prism software. Statistical differences in multiple groups were determined by multiple comparisons with ANOVA followed by Tukey post hoc tests. Statistical differences between 2 groups were determined by 2-tailed unpaired or paired Student t-test. *P* < 0.05 was considered significantly different.

## Supplementary Material

Supplemental Material
