# Supplementary material for: Clearance of damaged mitochondria via mitophagy is important to the protective effect of ischemic preconditioning in kidneys
Source: Autophagy. 2019 May 22;15(12):2142–62. doi: 10.1080/15548627.2019.1615822 (PMC6844514; doi:10.1080/15548627.2019.1615822)
Supplement: Supplemental Material [file kaup-15-12-1615822-s001.zip › Supplementary information/Supplemental Figures 20190305.pptx]

## Slide 1
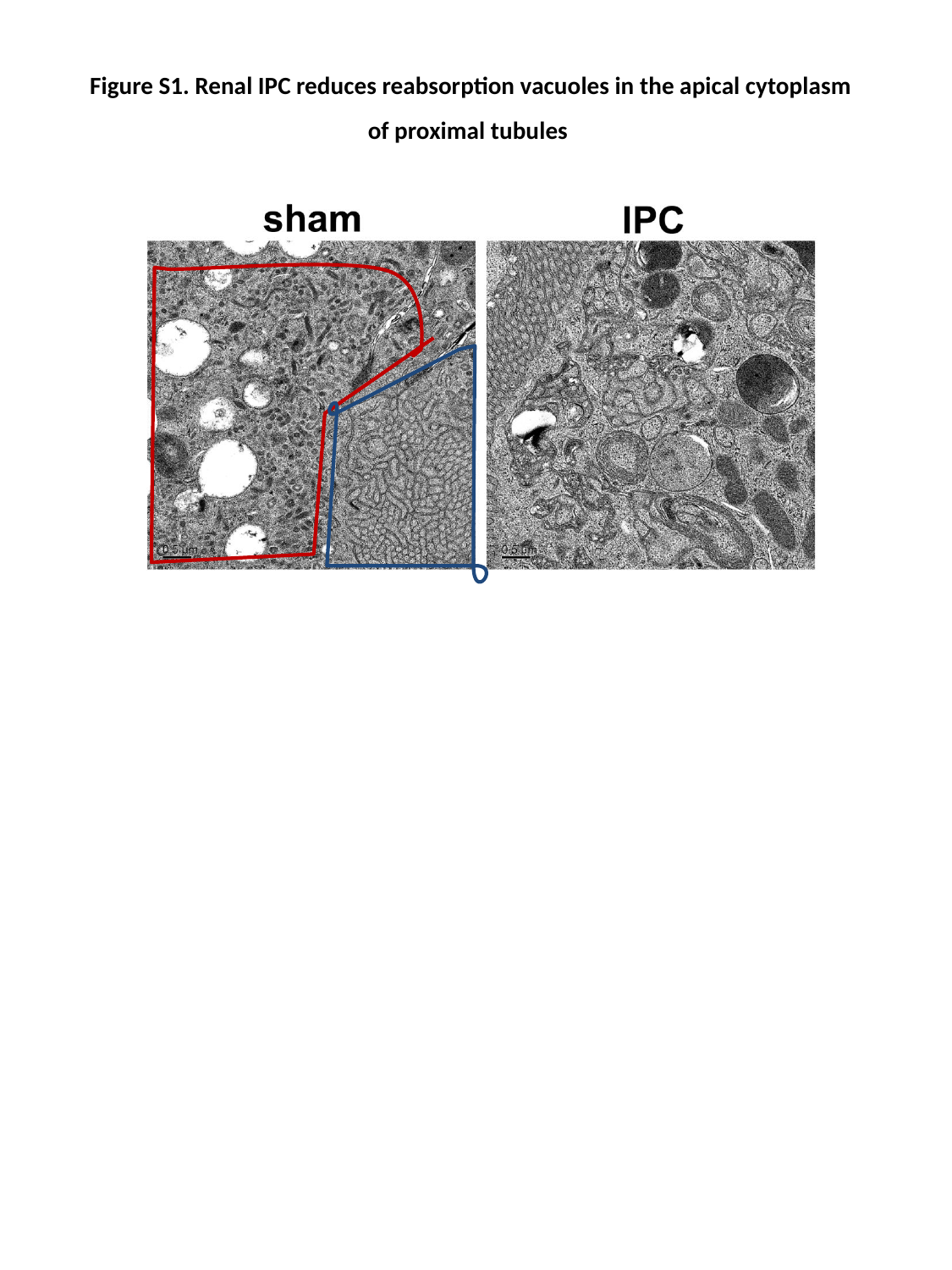

Figure S1. Renal IPC reduces reabsorption vacuoles in the apical cytoplasm of proximal tubules

## Slide 2
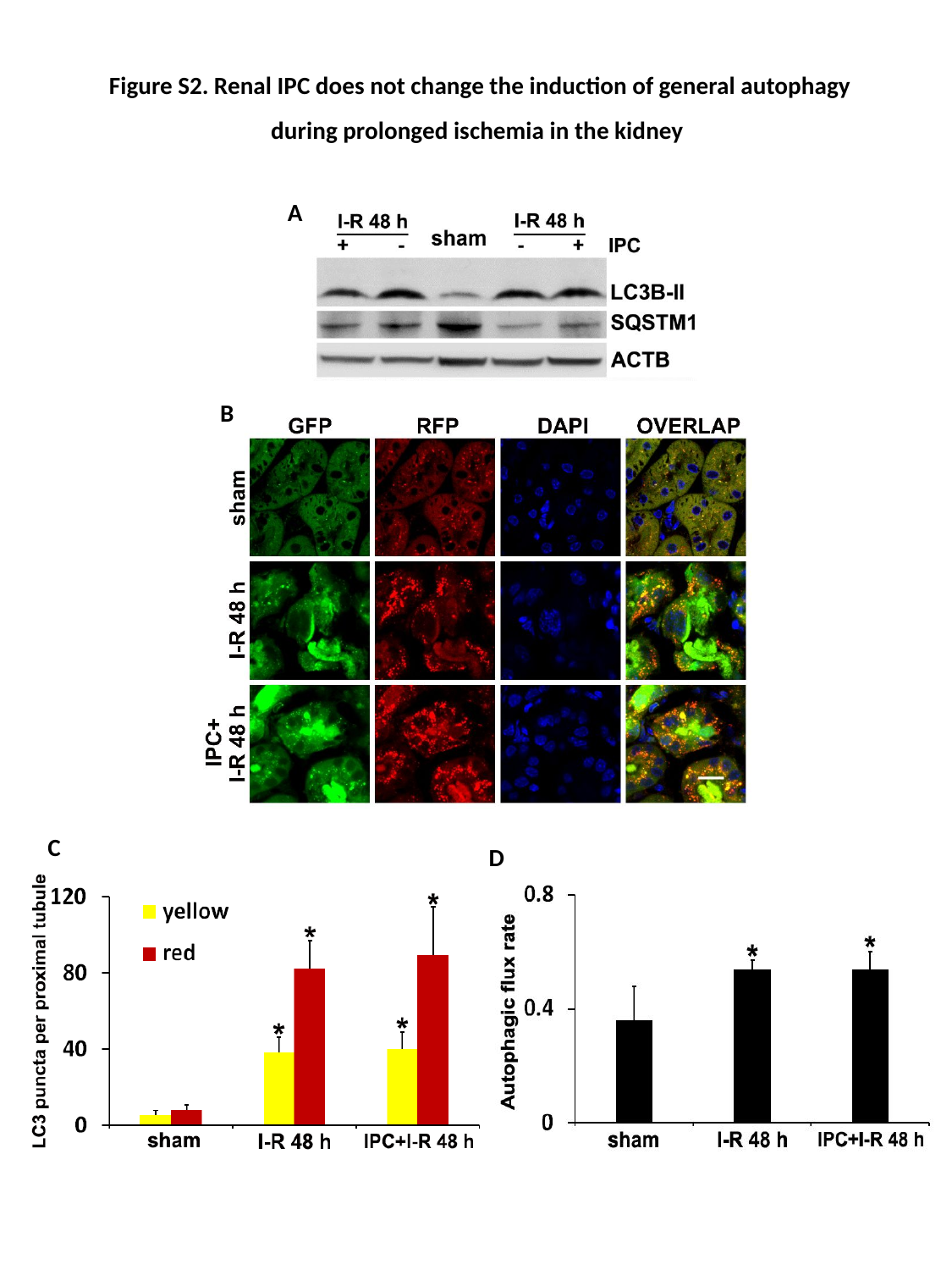

Figure S2. Renal IPC does not change the induction of general autophagy during prolonged ischemia in the kidney
A
B
C
D

## Slide 3
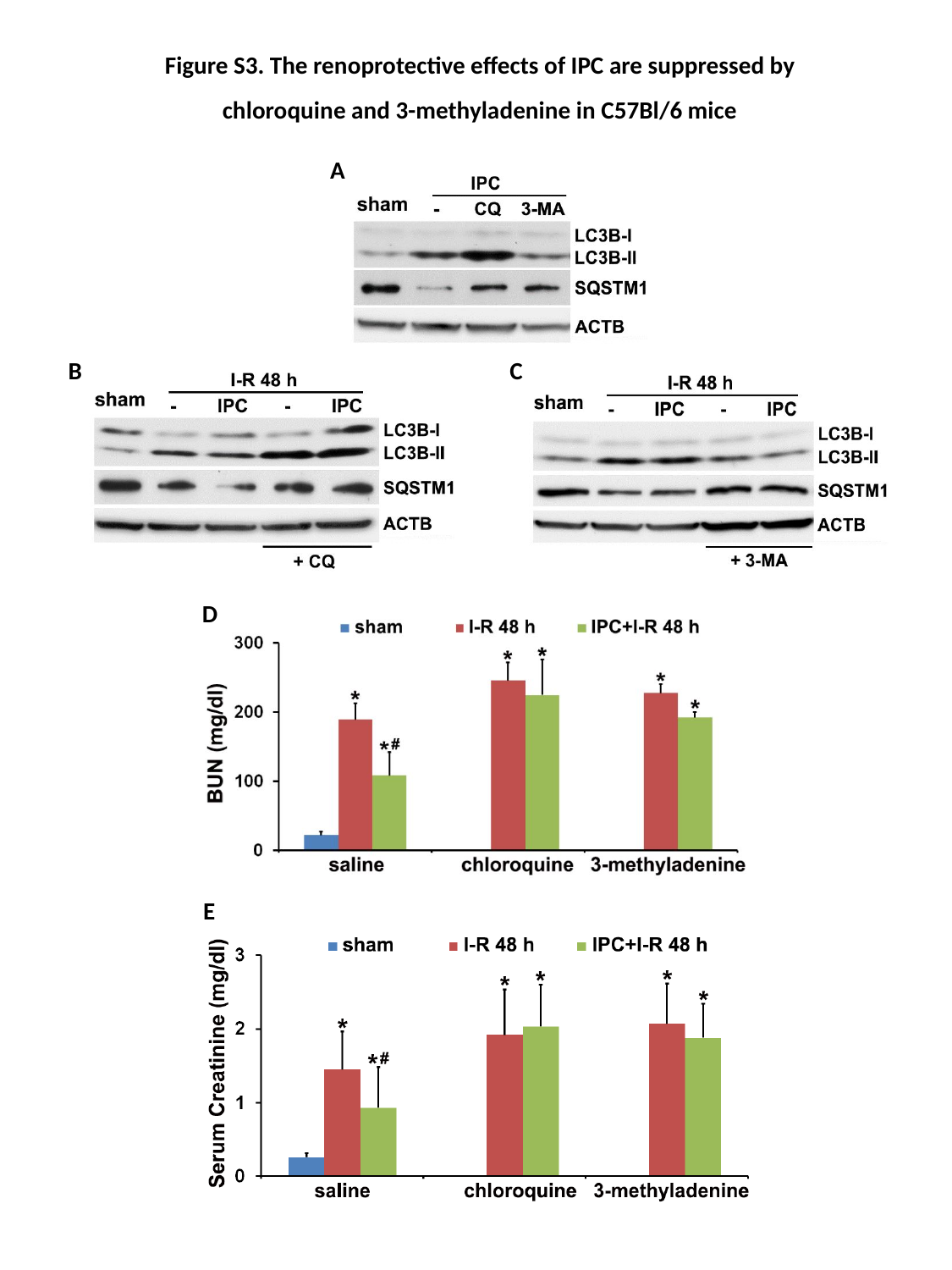

Figure S3. The renoprotective effects of IPC are suppressed by chloroquine and 3-methyladenine in C57Bl/6 mice
A
B
C
D
E

## Slide 4
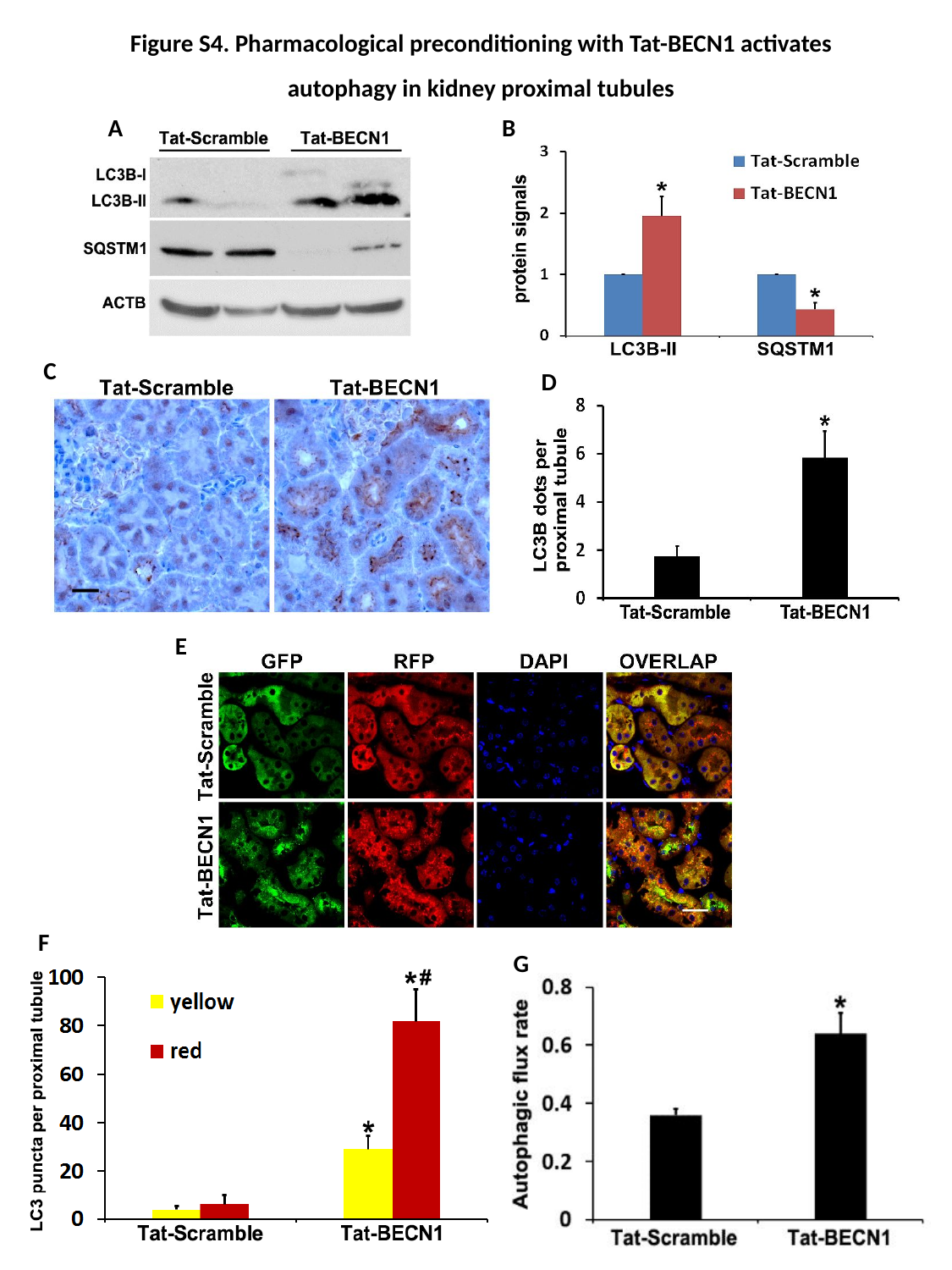

Figure S4. Pharmacological preconditioning with Tat-BECN1 activates autophagy in kidney proximal tubules
A
B
C
D
E
F
G

## Slide 5
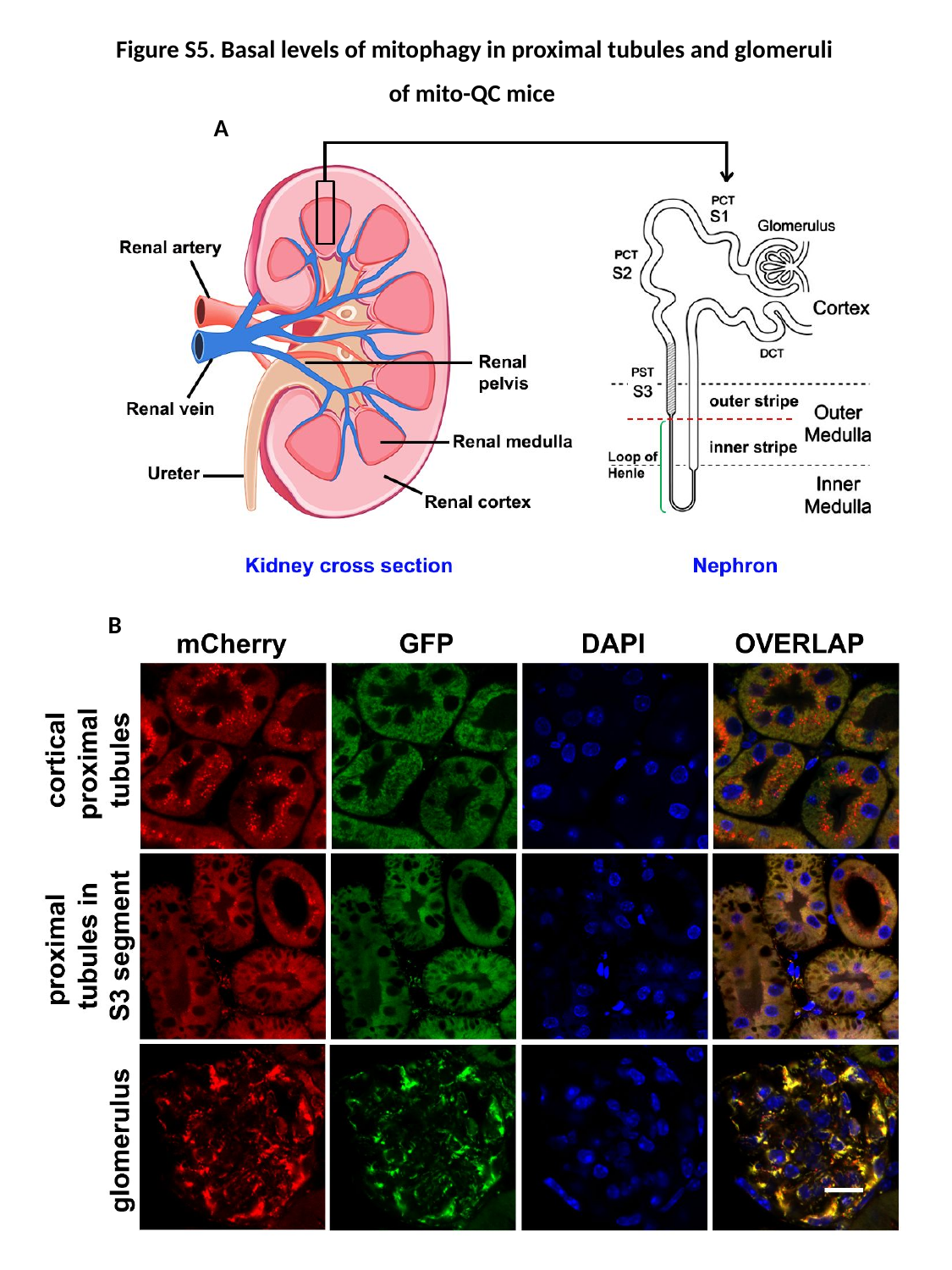

Figure S5. Basal levels of mitophagy in proximal tubules and glomeruli of mito-QC mice
A
B

## Slide 6
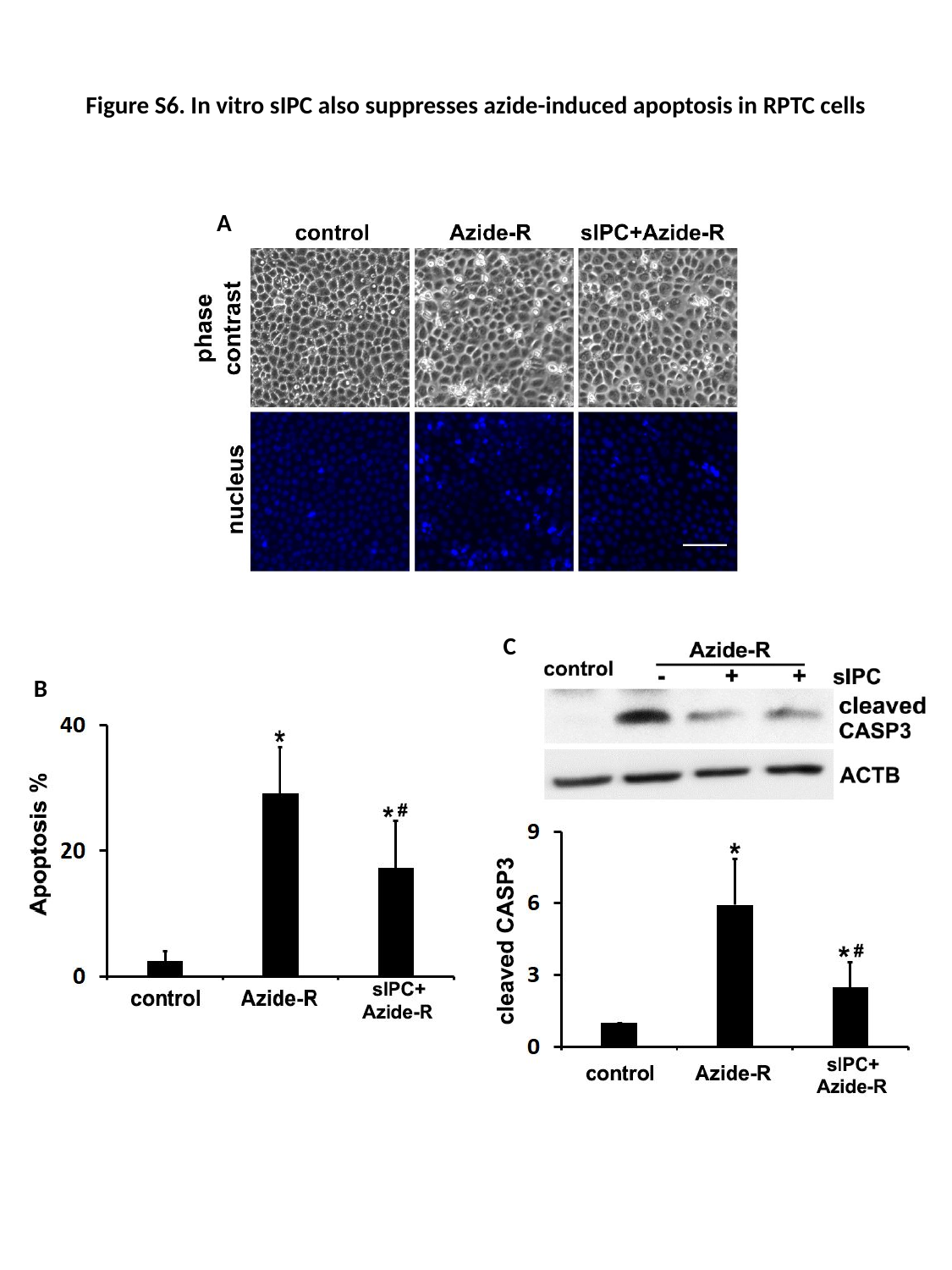

Figure S6. In vitro sIPC also suppresses azide-induced apoptosis in RPTC cells
A
C
B

## Slide 7
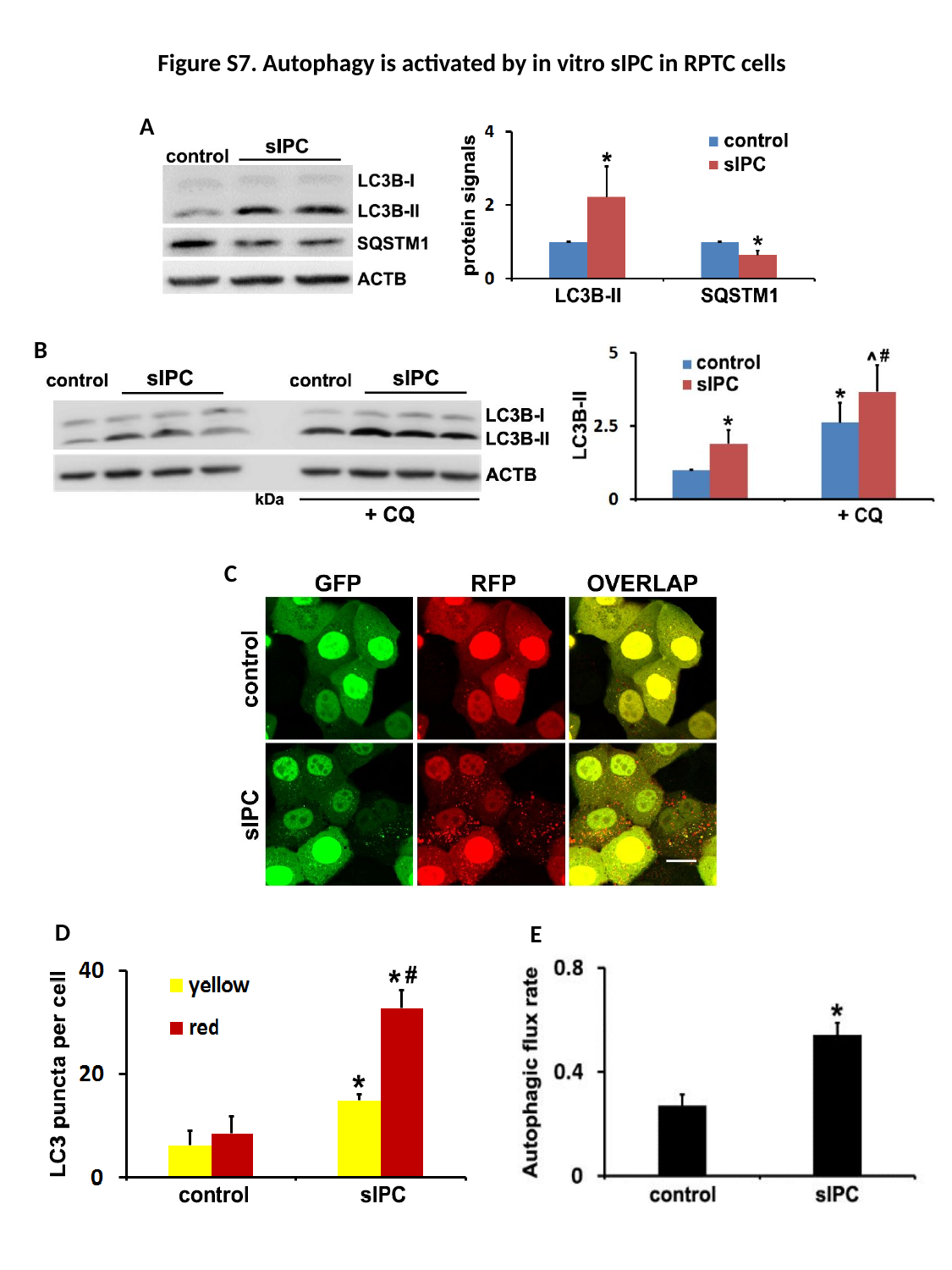

Figure S7. Autophagy is activated by in vitro sIPC in RPTC cells
A
B
C
D
E

## Slide 8
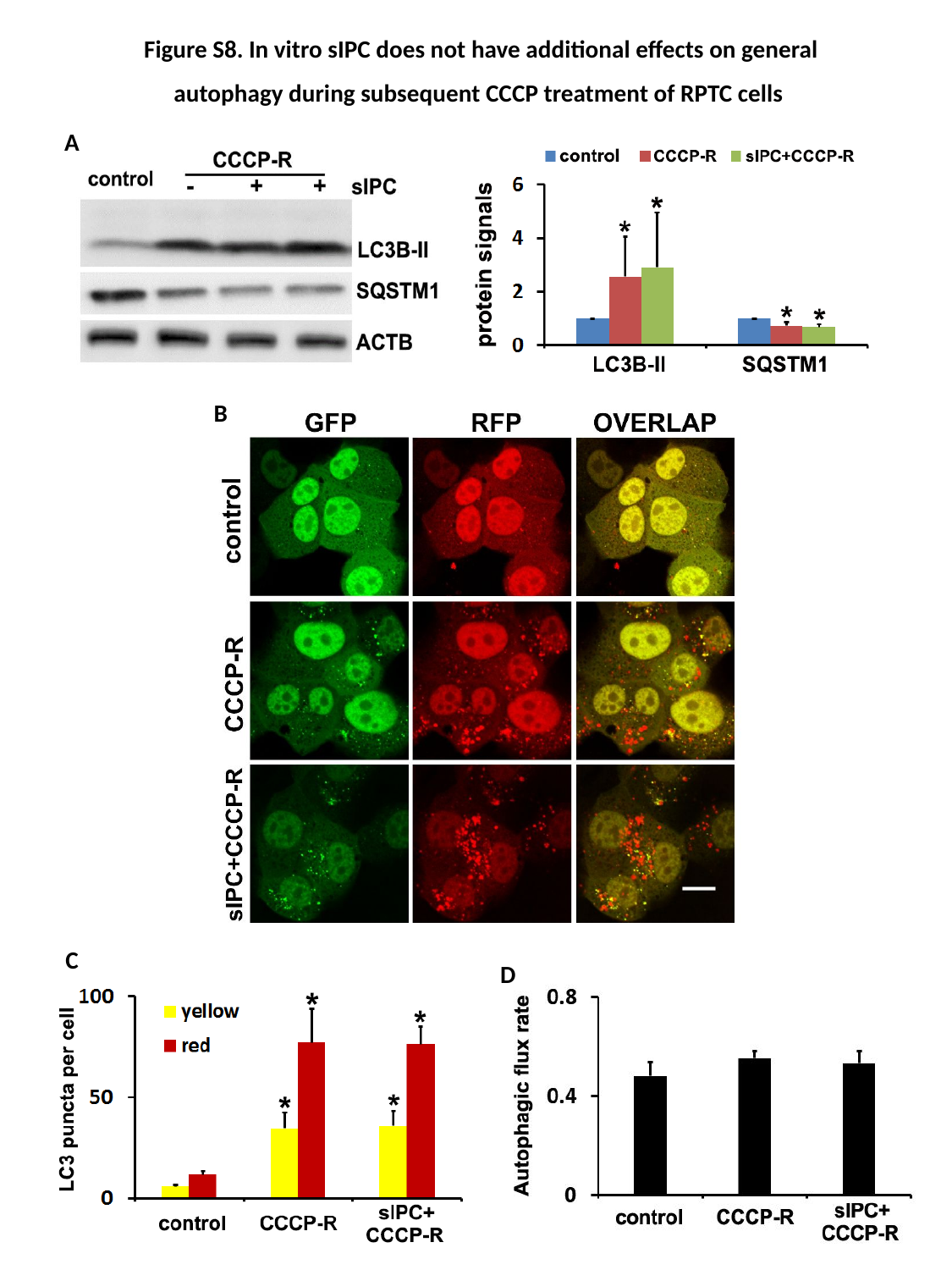

Figure S8. In vitro sIPC does not have additional effects on general autophagy during subsequent CCCP treatment of RPTC cells
A
B
C
D

## Slide 9
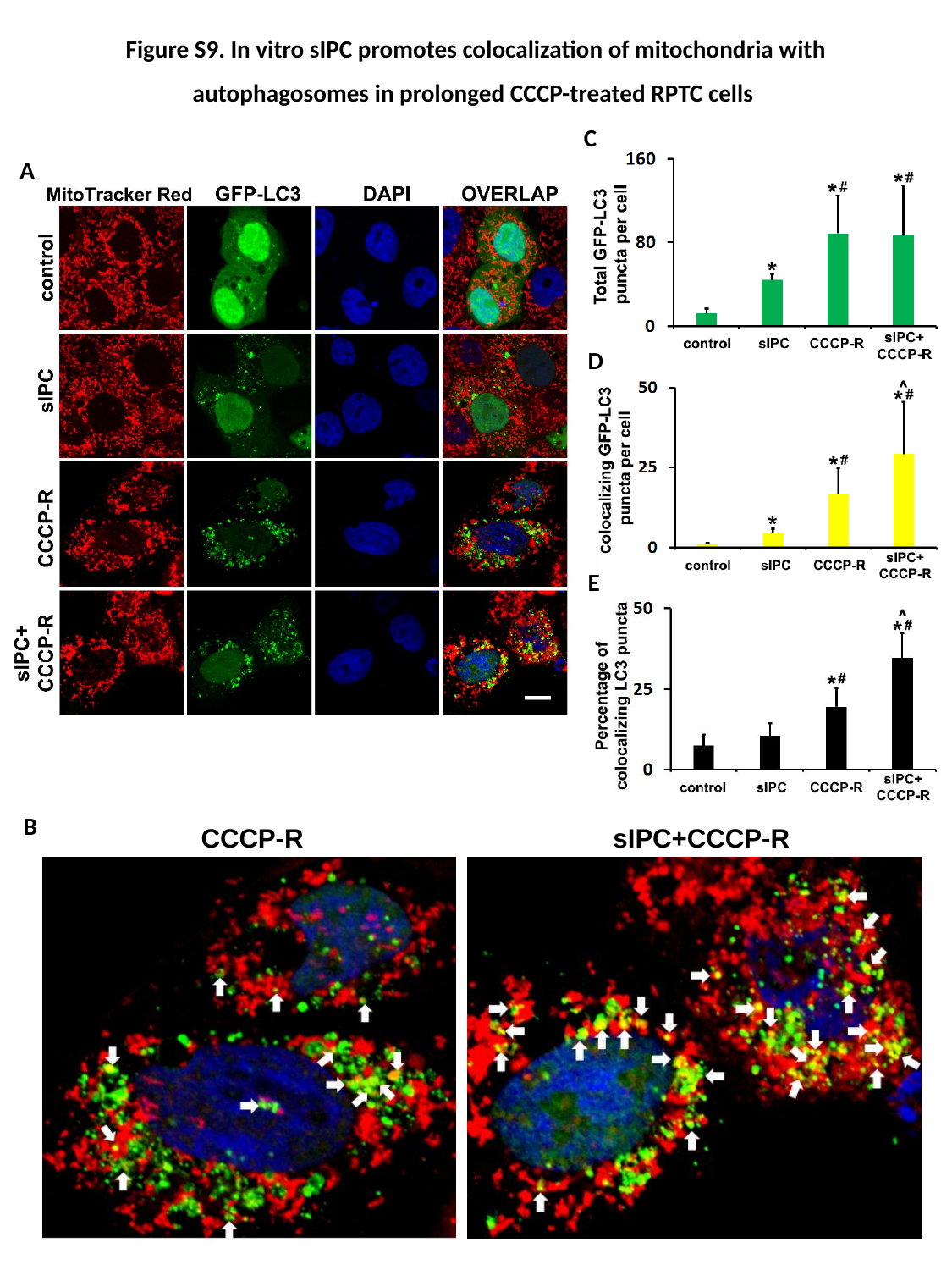

Figure S9. In vitro sIPC promotes colocalization of mitochondria with autophagosomes in prolonged CCCP-treated RPTC cells
C
A
D
E
B
CCCP-R
sIPC+CCCP-R

## Slide 10
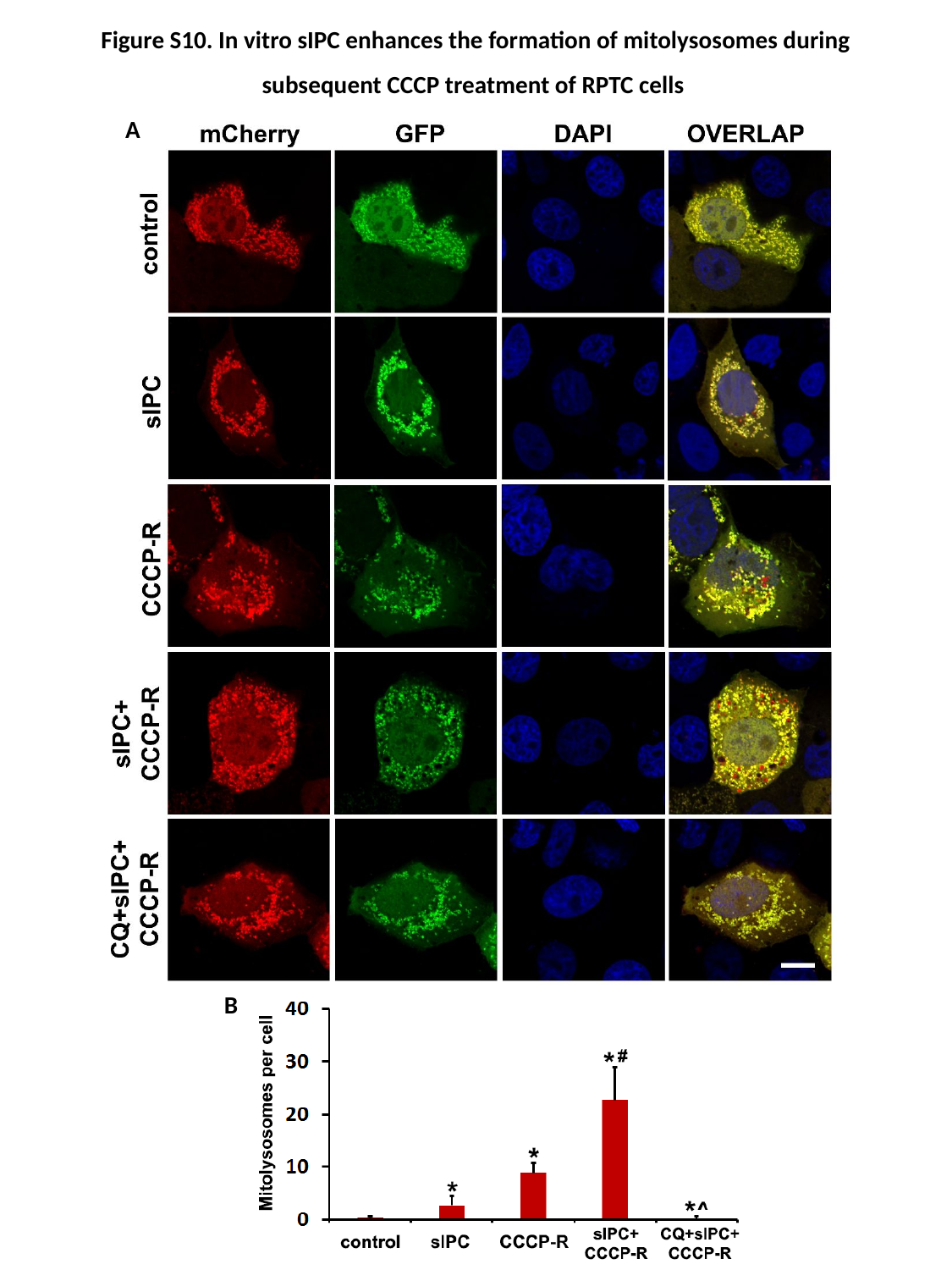

Figure S10. In vitro sIPC enhances the formation of mitolysosomes during subsequent CCCP treatment of RPTC cells
A
B

## Slide 11
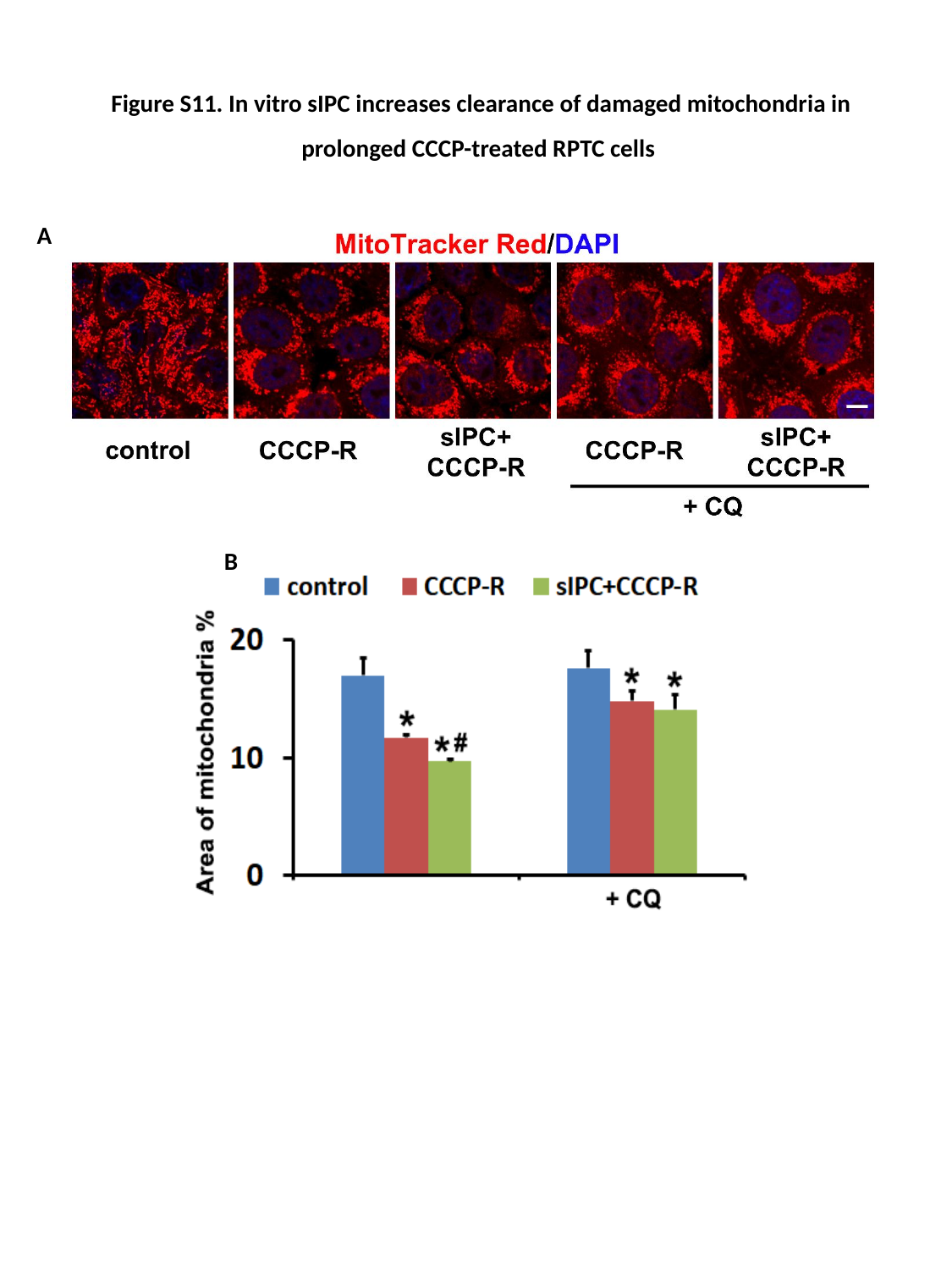

Figure S11. In vitro sIPC increases clearance of damaged mitochondria in prolonged CCCP-treated RPTC cells
A
B
