# Supplementary material for: Clearance of damaged mitochondria via mitophagy is important to the protective effect of ischemic preconditioning in kidneys
Source: Autophagy. 2019 May 22;15(12):2142–62. doi: 10.1080/15548627.2019.1615822 (PMC6844514; doi:10.1080/15548627.2019.1615822)
Supplement: Supplemental Material [file kaup-15-12-1615822-s001.zip › Supplementary information/Supplementary caption.docx]

**Figure S1.** Renal IPC reduces reabsorption vacuoles in the apical cytoplasm of proximal tubules. C57BL/6 mice were subjected to sham or renal IPC and kidneys were collected for EM analysis (n=2 for each). Representative electron micrographs showed organelle distribution immediately beneath the microvilli (brush border for light microscopy). The blue circled area indicated microvilli and the red curve outlined the sub-microvilli compartment that contains many reabsorption vacuoles, including bright, large vacuoles and dense, small coated vesicles (pinocytotic vesicles). The number of reabsorption vacuoles in proximal tubules was remarkably reduced by renal IPC. Scale bar: 500 nm.

**Figure S2.** Renal IPC does not change the induction of general autophagy during prolonged ischemia in the kidney. C57BL/6 (**A**) and CAGp-RFP-GFP-LC3 (**B**-**D**) mice were subjected to: (1) sham; (2) I-R; (3) IPC + I-R. Kidneys were collected for histology and immunoblot analyses (C57BL/6 mice: sham: n=3; I-R: n=7; IPC + I-R: n=10; CAGp-RFP-GFP-LC3 mice: n=3 for each). (**A**) Representative blot of LC3B and SQSTM1. ACTB was used as a loading control. (**B**) Representative images of GFP-LC3 and RFP-LC3 fluorescence staining. Scale bar: 15 µm. (**C**) Quantitative analysis of yellow and red LC3 puncta. (**D**) Analysis of autophagic flux rate. Data in (**C** and **D**) are expressed as mean ± SD. *, *P* < 0.05, significantly different from the sham group**.**

**Figure S3.** The renoprotective effects of IPC are suppressed by chloroquine and 3-methyladenine in C57BL/6 mice. (**A**) Mice were subjected to sham or renal IPC in the absence or presence of chloroquine (CQ, 60 mg/kg, i.p.) or 3-methyladenine (3-MA, 30 mg/kg, i.p.) (sham: n=3; n=6 for IPC, IPC + CQ and IPC + 3-MA groups). Both inhibitors were given 2 injections (1 d and 1 h) prior to IPC. Kidneys were collected for immunoblot analysis of LC3B and SQSTM1. ACTB was used as a loading control. (**B** and **C**) Mice were subjected to: (1) sham; (2) I-R; (3) IPC + I-R in the absence or presence of chloroquine or 3-methyladenine (sham: n=3; n=4 for I-R and IPC + I-R groups; n=6 for I-R + CQ, IPC + I-R + CQ, I-R + 3-MA and IPC + I-R + 3-MA groups). Both inhibitors were given 2 injections (1 d and 1 h) prior to ischemia and then daily during reperfusion. Kidneys were collected for immunoblot analysis of LC3B and SQSTM1. ACTB was used as a loading control. (**D** and **E**) Mice were treated as described in (**B** and **C**). Blood samples were collected for the measurements of BUN and serum creatinine. Data are expressed as mean ± SD. *, *P* < 0.05, significantly different from the sham group; #, *P* < 0.05, significantly different from I-R group.

**Figure S4.** Pharmacological preconditioning with Tat-BECN1 activates autophagy in kidney proximal tubules. C57BL/6 (**A**-**D**) and CAGp-RFP-GFP-LC3 (**E**-**G**) mice were given Tat-BECN1 and its control peptide (Tat-Scramble) at a single dose of 20 mg/kg i.p. injection (C57BL/6 mice: n=4 for each; CAGp-RFP-GFP-LC mice: n=3 for each). Four h after preconditioning, mice were sacrificed and kidneys were collected to determine autophagy induction. (**A**) Representative blots of LC3B and SQSTM1. ACTB was used as a loading control. (**B**) Densitometric analysis of LC3B-II and SQSTM1. After normalization with ACTB, the protein signals of the Tat-Scramble were arbitrarily set as 1, and the signals of Tat-BECN1 were normalized to the Tat-Scramble to calculate fold changes. (**C**) Representative images of immunohistochemical staining of LC3B. Scale bar: 20 µm. (**D**) Quantitative analysis of punctate LC3B staining. Data in (**B** and **D**) are expressed as mean ± SD. *, *P* < 0.05, significantly different from the Tat-Scramble group. (**E**) Representative images of GFP-LC3 and RFP-LC3 fluorescence staining. Scale bar: 15 µm. (**F**) Quantitative analysis of yellow and red LC3 puncta. Data are expressed as mean ± SD. *, *P* < 0.05, significantly different from the Tat-Scramble group; #, *P* < 0.05, values of red LC3 puncta significantly different from the relevant values of yellow LC3 puncta. (**G**) Analysis of autophagic flux rate. Data are expressed as mean ± SD. *, *P* < 0.05, significantly different from the Tat-Scramble group**.**

**Figure S5.** Basal levels of mitophagy in proximal tubules and glomeruli of mito-QC mice. (**A**) Schematic diagram of the kidney and a nephron (modified from online images: <https://www.ontariorenalnetwork.ca/en/kidney-care-resources/living-with-chronic-kidney-disease/about-chronic-kidney-disease?redirect=true#.WYDM2ITyuM8>; <https://www.oatext.com/The-effects-of-anesthesia-and-fetal-surgery-on-the-early-ovine-fetus.php>). The kidney is made up of renal lobes. The cortical components of the lobes are fused so that the renal cortex forms a continuous smooth outer zone which extend down between the renal medulla (medullary pyramids). Nephrons are the structural and functional units of the kidney, which arise in the cortex, loop down into the medulla and return to the cortex. The nephron consists of two major components, the renal corpuscle and the renal tubule. The renal corpuscle is a combination of two structures: Bowman’s capsule and the glomerulus. The renal tubule extends from Bowman’s capsule and has four distinct zones: the proximal tubule, the loop of Henle, the distal convoluted tubule (DCT), and the collecting tubule, each of which has a different role in tubular function and a corresponding difference in histological appearance. The proximal tubule (proximal convoluted tubule (PCT) and proximal straight tubule (PST)) is the longest section of the tubule and is responsible for the reabsorption of 65% of the ions and water of the glomerular filtrate. While the PCT is confined to the renal cortex and makes up the first (S1) and the second (S2) segments of the proximal tubule, the PST (S3 segment) is mainly located in the inner cortex and the outer stripe of outer medulla. (**B**) Kidneys from sham mito-QC mice were collected for the examination of basal mitophagy by fluorescence microscopy. Representative images showed a high basal level of mitophagy in a portion of cortical proximal tubules, with mitolysosomes (red-only puncta) localized predominantly at the apical side toward the lumen. By contrast, proximal tubules at S3 segment had minimal mitochondrial turnover under control condition. In glomeruli scattered mitolysosomes were detected in sham mice. Scale bar: 15 µm.

**Figure S6.** In vitro sIPC also suppresses azide-induced apoptosis in RPTC cells. In vitro sIPC were induced by incubating RPTC cells with 10 mM sodium azide for 30 min followed by 40 min of recovery. The cells were then treated with prolonged azide (10 mM) for 3 h followed by 2 h of recovery to model in vivo renal IRI. Cells were collected for analysis of apoptosis by morphology and caspase activation. (**A**) Representative images of phase contrast and fluorescence microscopy showing cellular and nuclear morphology of apoptosis. Scale bar: 200 μm. (**B**) Quantification of cell apoptosis. (**C**) Representative blots and densitometric analysis of cleaved CASP3. ACTB was used as a loading control. After normalization with ACTB, the protein signal of the control was arbitrarily set as 1, and the signals of other conditions were normalized to the control to calculate fold changes. Data in (**B** and **C**) are expressed as mean ± SD. *, *P* < 0.05, significantly different from the control group; #, *P* < 0.05, significantly different from Azide-R group.

**Figure S7.** Autophagy is activated by in vitro sIPC in RPTC cells. (**A**) RPTC cells were untreated (control) or treated with sIPC. Cells were collected for immunoblot analysis of LC3B and SQSTM1. (**B**) RPTC cells were treated with sIPC in the absence or presence of chloroquine (CQ, 20 μM). Cells were collected for immunoblot analysis of LC3B. ACTB was used as a loading control. The molecular mass marker lane was labelled as kDa. For densitometric analysis in (**A** and **B**), after normalization with ACTB, the protein signals of the control were arbitrarily set as 1, and the signals of other conditions were normalized to the control to calculate fold changes. Data are expressed as mean ± SD. *, *P* < 0.05, significantly different from the control group; #, *P* < 0.05, significantly different from sIPC group; ^, significantly different from control + CQ group. RPTC cells were transiently transfected with mRFP-GFP-LC3 and then untreated (control) or treated with sIPC. Cells were collected for fluorescence microscopy. (**C**) Representative images of GFP-LC3 and RFP-LC3 fluorescence staining. Scale bar: 15 µm. (**D**) Quantitative analysis of yellow and red LC3 puncta. Data are expressed as mean ± SD. *, *P* < 0.05, significantly different from the control group; #, *P* < 0.05, values of red LC3 puncta significantly different from the relevant values of yellow LC3 puncta. (**E**) Analysis of autophagic flux rate. Data are expressed as mean ± SD. *, *P* < 0.05, significantly different from the control group**.**

**Figure S8.** In vitro sIPC does not have additional effects on general autophagy during subsequent CCCP treatment of RPTC cells. (**A**) RPTC cells were subjected to: (1) control; (2) CCCP-R; (3) sIPC + CCCP-R. Cells were collected for immunoblot analysis of LC3B and SQSTM1. ACTB was used as a loading control. For densitometric analysis, after normalization with ACTB, the protein signals of the control were arbitrarily set as 1, and the signals of other conditions were normalized to the control to calculate fold changes. Data are expressed as mean ± SD. *, *P* < 0.05, significantly different from the control group. RPTC cells were transiently transfected with mRFP-GFP-LC3 and then subjected to the treatment as described in (**A**). Cells were collected for fluorescence microscopy. (**B**) Representative images of GFP-LC3 and RFP-LC3 fluorescence staining. Scale bar: 15 µm. (**C**) Quantitative analysis of yellow and red LC3 puncta. (**D**) Analysis of autophagic flux rate. Data in (**C** and **D**) are expressed as mean ± SD. *, *P* < 0.05, significantly different from the control group.

**Figure S9.** In vitro sIPC promotes colocalization of mitochondria with autophagosomes in prolonged CCCP-treated RPTC cells. RPTC cells were transiently transfected with GFP-LC3 for 24 h and then incubated with MitoTracker Red CMXRos (50 nM) for 30 min. The cells were then subjected to: (1) control; (2) sIPC; (3) CCCP-R; (4) sIPC + CCCP-R in the presence of chloroquine. Cells were collected for fluorescence microscopy. (**A**) Representative images of mitochondria and GFP-LC3 puncta. Scale bar: 10 μm. (**B**) Enlarged images showing the colocalization of mitochondria and autophagosomes in detail (arrows). (**C**) Quantification of total GFP-LC3 puncta. (**D**) Quantification of colocalizing GFP-LC3 puncta. (**E**) Percentage of colocalizing GFP-LC3 puncta in total GFP-LC3 puncta. Data in (**C**, **D** and **E**) are expressed as mean ± SD. *, *P* < 0.05, significantly different from the control group; #, *P* < 0.05, significantly different from sIPC group; ^, *P* < 0.05, significantly different from CCCP-R group.

**Figure S10.** In vitro sIPC enhances the formation of mitolysosomes during subsequent CCCP treatment of RPTC cells. RPTC cells were transiently transfected with COX8-EGFP-mCherry for 24 h and then subjected to: (1) control; (2) sIPC; (3) CCCP-R; (4) sIPC + CCCP-R; (5) CQ + sIPC + CCCP-R. Cells were collected for fluorescence microscopy. (**A**) Representative images of mitolysosome formation. Scale bar: 10 μm. (**B**) Quantitative analysis of the number of mitolysosomes per cell. Data are expressed as mean ± SD. *, *P* < 0.05, significantly different from the control group; #, *P* < 0.05, significantly different from CCCP-R group; ^, *P* < 0.05, significantly different from sIPC + CCCP-R group.

**Figure S11.** In vitro sIPC increases clearance of damaged mitochondria in prolonged CCCP-treated RPTC cells. RPTC cells were incubated with 50 nM MitoTracker Red CMXRos for 30 min and then treated with: (1) control; (2) CCCP-R; (3) sIPC + CCCP-R in the absence or presence of chloroquine (CQ). Cells were collected for fluorescence microscopy. (**A**) Representative images of MitoTracker Red staining showing mitochondrial morphology. Scale bar: 10 μm. (**B**) Quantification of the amount of remaining mitochondria. Data are expressed as mean ± SD. *, *P* < 0.05, significantly different from the control group; #, *P* < 0.05, significantly different from CCCP-R group.
